# Derivation and Expansion Using Only Small Molecules of Human Neural Progenitors for Neurodegenerative Disease Modeling

**DOI:** 10.1371/journal.pone.0059252

**Published:** 2013-03-22

**Authors:** Peter Reinhardt, Michael Glatza, Kathrin Hemmer, Yaroslav Tsytsyura, Cora S. Thiel, Susanne Höing, Sören Moritz, Juan A. Parga, Lydia Wagner, Jan M. Bruder, Guangming Wu, Benjamin Schmid, Albrecht Röpke, Jürgen Klingauf, Jens C. Schwamborn, Thomas Gasser, Hans R. Schöler, Jared Sterneckert

**Affiliations:** 1 Department of Cell and Developmental Biology, Max Planck Institute for Molecular Biomedicine, Münster, North Rhine Westphalia, Germany; 2 Stem Cell Biology and Regeneration Group, Institute of Cell Biology, Center for Molecular Biology of Inflammation, Westfälische Wilhelms-Universität Münster, Münster, North Rhine-Westphalia, Germany; 3 Westfälische Wilhelms-Universität Münster, Institute for Medical Physics and Biophysics, Cellular Biophysics Group, Münster, North Rhine-Westphalia, Germany; 4 Center for Research in Molecular Medicine and Chronic Diseases at the University of Santiago de Compostela, Santiago de Compostela, Spain; 5 Department of Neurodegenerative Diseases, Hertie-Institute for Clinical Brain Research, University of Tübingen, and German Center for Neurodegenerative Diseases, Tübingen, Baden-Württemburg, Germany; 6 Institute for Human Genetics, University of Münster, Münster, North Rhine Westphalia, Germany; 7 Medical Faculty, University of Münster, Münster, North Rhine-Westphalia, Germany; Stanford University School of Medicine, United States of America

## Abstract

Phenotypic drug discovery requires billions of cells for high-throughput screening (HTS) campaigns. Because up to several million different small molecules will be tested in a single HTS campaign, even small variability within the cell populations for screening could easily invalidate an entire campaign. Neurodegenerative assays are particularly challenging because neurons are post-mitotic and cannot be expanded for implementation in HTS. Therefore, HTS for neuroprotective compounds requires a cell type that is robustly expandable and able to differentiate into all of the neuronal subtypes involved in disease pathogenesis. Here, we report the derivation and propagation using only small molecules of human neural progenitor cells (small molecule neural precursor cells; smNPCs). smNPCs are robust, exhibit immortal expansion, and do not require cumbersome manual culture and selection steps. We demonstrate that smNPCs have the potential to clonally and efficiently differentiate into neural tube lineages, including motor neurons (MNs) and midbrain dopaminergic neurons (mDANs) as well as neural crest lineages, including peripheral neurons and mesenchymal cells. These properties are so far only matched by pluripotent stem cells. Finally, to demonstrate the usefulness of smNPCs we show that mDANs differentiated from smNPCs with *LRRK2* G2019S are more susceptible to apoptosis in the presence of oxidative stress compared to wild-type. Therefore, smNPCs are a powerful biological tool with properties that are optimal for large-scale disease modeling, phenotypic screening, and studies of early human development.

## Introduction

Stem cells are positioned to revolutionize drug discovery for neurodegenerative disorders through *in vitro* disease modeling and HTS. Through reprogramming of primary cells from a patient, induced pluripotent stem cells (iPSCs) can be generated with properties comparable to human embryonic stem cells (hESCs) [1]. For example, two groups have reported that midbrain dopaminergic neurons (mDANs) differentiated from iPSCs generated from patients with Parkinson’s disease (PD) with mutations in the gene LRRK2 exhibit disease-associated phenotypes [2], [3]. Similarly, it has been demonstrated that motor neurons (MNs) differentiated from hESCs with mutations causing amyotrophic lateral sclerosis (ALS) are susceptible to degeneration [4]. In principle, the phenotypes exhibited by patient-specific cells could be used in high-throughput screening (HTS) campaigns to identify novel neuroprotective compounds for development into new drugs.

Because HTS campaigns can involve up to several million independent wells, the reproducibility of the assay must be extremely high, or else the results will be uninterpretable. Consequently, for the promise of stem cells to become realized at least two hurdles must be overcome. First, HTS using models of neurodegenerative diseases requires cells that are characterized by very robust expansion to produce billions of cells under chemically defined conditions. Second, these cells must be capable of efficient formation of neurons such as mDANs and MNs. However, current stem cell protocols are not robust, require expensive recombinant growth factors, involve manual manipulation, have need of frequent splitting at narrow ratios, need significant time for differentiation, and/or result in inefficient differentiation. As a result, stem cell cultures typically exhibit tremendous variability and, as a result, it is often difficult to reproducibly obtain a statistically significant result using triplicate wells. Therefore, previous cell types including neural stem cells (NSCs) [5], long-term self-renewing rosette-type hESC-derived NSCs (lt-hESNSCs) [6], primitive NSCs (pNSCs) [7], and rosette neural cells (R-NCs) [8], are not easily compatible with HTS. We have identified a novel type of neural progenitor cells capable of robust, immortal expansion and efficient differentiation into both central nervous system (CNS) and neural crest lineages. These properties are so far only matched by pluripotent stem cells. Additionally, our neural precursor cells only require small molecules for self-renewal and expansion, a feature that significantly reduces the cost of large-scale disease modeling and, to date, is not possible with any other available cell type.

Although NSCs are competent to differentiate into CNS lineages including neurons, astrocytes and oligodendrocytes, they are not able to efficiently form mDANs or MNs, which makes them unsuitable for neurodegenerative disease modeling [5], [9]. Similar to NSCs, lt-hESNSCs, which are differentiated from hESCs, use the same recombinant proteins as NSCs for self-renewal – FGF2 and EGF – and express markers of ventral hindbrain identity. When treated with the developmental patterning factors Sonic hedgehog (SHH) and Fibroblast growth factor 8 (FGF8) or SHH and retinoic acid (RA), about 70% of differentiated neurons were not mDANs. When cultured with SHH and retinoic acid (RA), about 85% of differentiated neurons were not MNs. These results make large-scale modelling of PD and ALS problematic. In addition, lt-hESNSCs require splitting three or more times per week at very low ratios, which makes expansion to billions of cells for HTS very tedious and cumbersome.

Two cell types have been reported with increased differentiation potential. Primitive NSCs (pNSCs) could efficiently be differentiated into mDANs (about 55%) and MNs (about 54%). However, pNSCs require recombinant Leukemia inhibitory factor (LIF) for self-renewal, which makes them very cost-intensive to culture in large quantities and, therefore, impractical for HTS. Elkabetz *et alia* reported the derivation of rosette-neural cells (R-NCs) from hESCs [8]. R-NCs could be differentiated into both CNS and neural crest lineages. However, despite the use of high doses of recombinant growth factors, R-NCs were only expandable for up to 4 passages before differentiating. As a result, it is not possible to generate enough R-NCs for even a pilot study to validate an HTS assay let alone an entire HTS campaign. As a result, it is urgently necessary to derive a cell type that is both plastic and can be propagated in a manner compatible with HTS.

We speculated that signals present in the neural plate border region may be sufficient to direct self-renewal of cells *in vitro* with potential to differentiate into CNS and neural crest lineages including peripheral nervous system (PNS) neurons. During embryogenesis, neuroepithelial cells on the border between the neural plate and neural crest have the developmental potential to form neural tube-derived CNS lineages as well as neural crest-derived lineages such as peripheral nervous system (PNS) neurons [10], [11]. WNT proteins specify formation of cells at the lateral border of the neural plate and are potent mitogens [12]. Patterning by WNT proteins is antagonized by SHH, which specifies ventral neural tube fates and is also a potent mitogen [12]. It is significant to note that SHH signaling was used by Elkabetz *et alia* to culture R-NCs [8]. Because R-NCs could not be expanded without committing to the CNS, we hypothesized that WNT signaling in combination with SHH signaling might contribute to the maintenance of precursors with developmental potential for both neural crest and neural plate.

Here we report the derivation of human smNPCs, which have properties uniquely suited to modeling neurodegenerative diseases. We show that smNPCs can be efficiently specified into neural tube and neural crest lineages. This developmental potential is similar to R-NCs, and developmentally upstream of NSCs, lt-hESNSCs, and pNSCs. Unlike R-NCs, we demonstrate that smNPCs are capable of immortal self-renewal using WNT and SHH signals. This combination is also distinct from FGF2 and EGF required for NSCs and lt-hESNSCs. We show that culturing smNPCs with FGF2 results in the formation of rosette-like structures, which have been previously associated with the neural plate-stage of embryogenesis [13]. In addition, smNPCs are easy to handle, do not require manual manipulation, and can be cultured at a wide range of cell densities using only inexpensive small molecules. Finally, we show that mDANs differentiated from smNPCs with the PD-associated mutation *LRRK2* G2019S are more sensitive to stress compared to wild-type. Therefore, smNPCs are a robust and affordable tool suitable for disease modeling.

## Results

### Derivation of a Population of Expandable Human Neural Epithelial Cells

**Figure 1:**
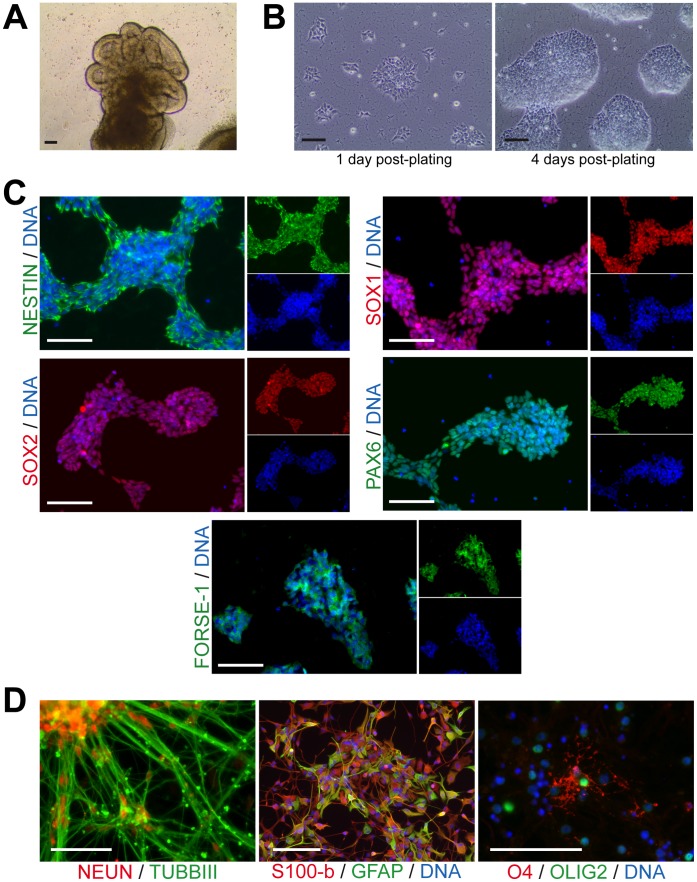
Derivation of neural epithelial cells. (A) Plated EBs differentiating in the presence of both PMA and CHIR for 6 days. (B) Phase-contrast images of neural epithelial cells on the indicated days after splitting. (C) Immunostaining of hESC-derived neural epithelial cells with antibodies raised against the indicated neural progenitor markers. Nuclei are counterstained with Hoechst. (D) Immunostaining of spontaneously differentiated neural epithelial cells for TUBBIII and NEUN, for GFAP and S100-beta after astrocyte differentiation, as well as O4 and OLIG2 after spontaneous differentiation, indicating oligodendrocyte formation. Scale bars are 100 µm. See also Figure S1.

We tested the effects of introducing both WNT and SHH signals to cultures of differentiating pluripotent stem cells. hESCs and hiPSCs were differentiated via human embryoid bodies (hEBs). Neural induction was initiated through inhibition of both BMP and TGFβ signaling using the small molecules dorsomorphin (DM) and SB43152 (SB) [14], [15]. The small molecule CHIR99021 (CHIR), a GSK3b inhibitor, was added to stimulate the canonical WNT signaling pathway. The SHH pathway was stimulated using the small molecule purmorphamine (PMA). Differentiating hEBs exposed to CHIR and PMA were marked by the formation and expansion of an epithelium (Figure 1A). These epithelial cells expressed markers of neural progenitors including SOX1, SOX2, NESTIN, and PAX6, but not mesodermal marker T (Figure S1A). When disaggregated and plated on Matrigel, homogeneous colonies of epithelial cells formed (Figure 1B). These neural epithelial cells could be split enzymatically without manual selection at a 1∶10 to 1∶20 ratio and expanded as cell lines for more than 150 population doublings and exhibited a diploid karyotype (Figure S1B). An analysis of doubling time indicated that neural epithelial cells divided approximately once per day, which was consistent over multiple passages as well as cultures derived from multiple pluripotent stem cell lines (Figure S1C). Immunostaining of neural epithelial cell colonies demonstrated the uniform expression of the neural progenitor markers SOX1, SOX2, NESTIN, and PAX6 (Figures 1C). Immunostaining also showed the expression of FORSE1, which has been previously associated with early neural progenitors (Figure 1C) [7]. Upon spontaneous differentiation by withdrawal of the small molecules used for expansion, smNPC differentiated into cells expressing NEUN/TUBBIII, GFAP/S100beta, and O4/OLIG2, which mark neurons, astrocytes, and oligodendrocytes (Figures 1D and S2). Therefore, neural epithelial cells express markers that are characteristic of early neural progenitors. Interestingly, the cellular morphology and culture conditions of the neural epithelial cells are not typical of NSCs [5].

Neural epithelial cells after 20 passages were further characterized by microarray expression analysis. These cells did not express the pluripotency markers *OCT4* and *NANOG*, the mesendodermal markers *AFP*, *T*, and *SOX17*, and the trophoblast marker *EOMES* (Figure S3A). In contrast, neural epithelial cells showed high expression of neural precursor markers, including *SOX2*, *PAX6*, *HES5*, and *ASCL1* (Figure S3A). qRT-PCR analysis confirmed that neural epithelial cells express markers of neural progenitors, including *SOX2*, *SOX1*, *PAX6*, and *PAX3*, which were stably expressed beginning at about passage 5 (Table S1 and Figure S3B). This suggests that neural epithelial cells stably expressed markers of neural fate commitment after extensive cell culture starting at about passage 4. qRT-PCR also confirmed that neural epithelial cells did not express non-neural markers, including *OCT4*, *AFP*, *SOX17*, *CK8*, *CK18*, and *T*, after 4 to 5 passages (Figure S3B). This was achieved without any manual manipulation, just by replating the cells under specific culture conditions that strongly favored the expansion of these cells. These results demonstrate that cultures of neural epithelial cells do not contain subpopulations of cells expressing genetic markers of mesodermal, endodermal, trophoblastic, or pluripotent cells.

Interestingly, although the neural epithelial cells do not morphologically resemble neural rosettes, microarray analysis demonstrated strong expression of the (pre-) neural rosette genetic markers *DACH1*, *PLZF*, *LMO3, NR2F1, PLAGL1, LIX1,* and *EVI1* (Figure S3A). This suggested that neural epithelial cells might have the ability to form neural rosettes. To test this, we cultured neural epithelial cell colonies in the presence of FGF2, which has previously been reported to induce hEBs to form neural rosettes [7], [16]. After 2 days of culturing neural epithelial cell colonies in the presence of FGF2, numerous areas were found to exhibit the morphology of neural rosettes (Figure S4A). For further characterization, we immunostained the neural epithelial cells for N-CADHERIN and ZO-1, which is expressed by neural rosettes but spatially localized to the apical surface [8]. Although N-CADHERIN and ZO-1 expression were readily detected in colonies of neural epithelial cells, they were not spatially localized within the colonies (Figure S4B). In contrast, after FGF2 treatment, N-CADHERIN and ZO-1 expression were found at the apical surface of the rosettes (Figure S4B). Therefore, neural epithelial cells express early rosette markers and are capable of forming neural rosettes when cultured under appropriate conditions.

### CHIR and PMA Modulate Expression of Markers of Neural Crest and Ventral Neural Tube Identity in Human Neural Epithelial Cells

Using microarray transcription profiling, we assessed the expression of markers of regional identity by cultures neural epithelial cells. *PAX3*, *IRX3* and *MSX1*, which mark marginal neural plate [17] or neural plate border cells [12], were readily detectable in neural epithelial cells (Figure S5A). *SOX9*, which marks neural crest cells, was also present, albeit to a lesser extent. *NKX6.1*, *OLIG2*, *NKX2.2*, and *FOXA2*, which mark medial neural plate and ventral neural tube, were not detected. *GSH2*, which marks dorsal neural tube progenitors, was also not detected. Of the tested rostrocaudal markers, only *HOXA2* and *HOXB2*, which mark anterior hindbrain identity, were highly expressed (Figure S5B). This pattern is consistent with the neural plate border and marginal neural plate region, which could suggest competence to differentiate into CNS and PNS lineages [10], [11], [12], [18]. However, we are unaware of cells *in vivo* that express the same combination of markers as smNPCs.

**Figure 2:**
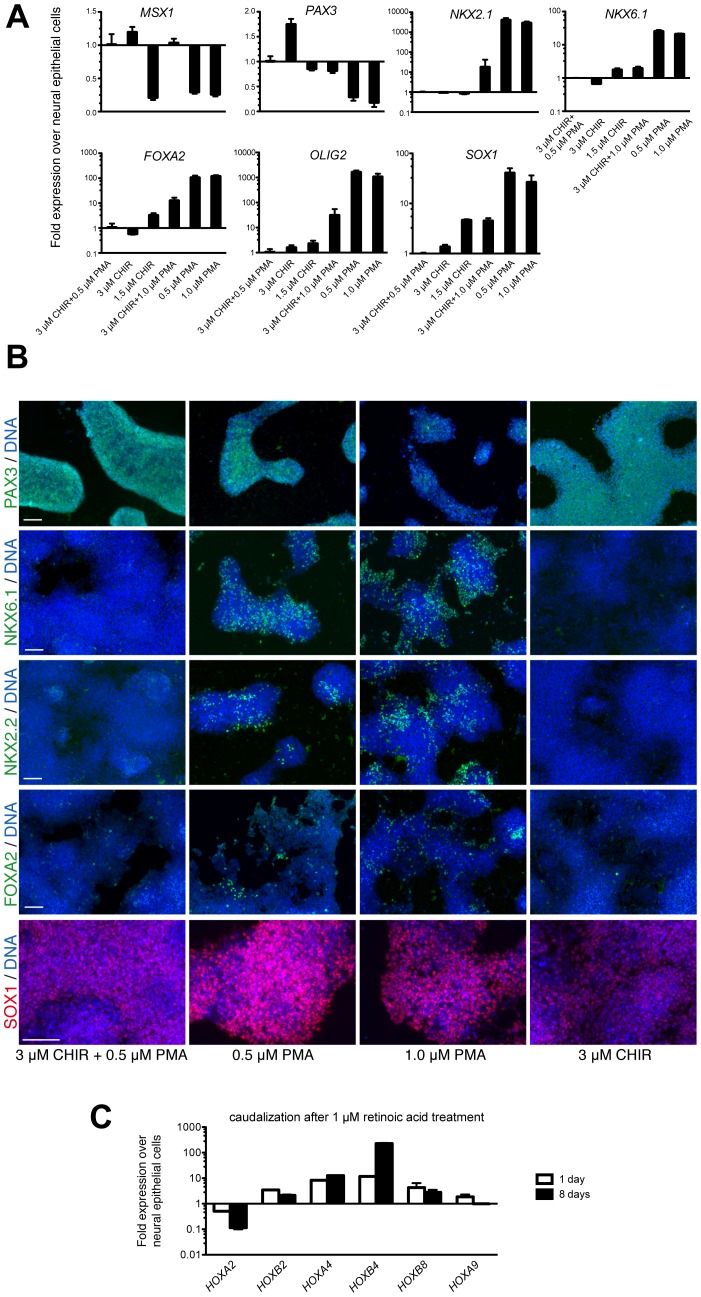
Neural epithelial cells can be respecified along both the dorsoventral and rostrocaudal axes. (A) qRT-PCR analysis for the indicated marker on neural epithelial cells cultured under the indicated conditions for 6 days. Error bars represent the standard deviation from 3 independent cultures. (B) Immunostaining of neural epithelial cells cultured with the indicated concentration of PMA or CHIR for 6 days for PAX3, NKX2.2, NKX6.1, FOXA2, and SOX1. (C) qRT-PCR analysis of neural epithelial cells cultured with 1 µM RA for 1 or 8 days for the indicated rostrocaudal marker. Error bars represent the variation from 2 independent cultures. Scale bars are 100 µm. See also Figure S3.

Next, we asked if altering the concentrations of CHIR and PMA effected expression of lineage markers by cultures of neural epithelial cells. To answer this, neural epithelial cells at passages 15–20 were cultured with different concentrations of CHIR and PMA alone or in combination for 6 days. qRT-PCR demonstrated that 3 µM CHIR alone induced expression of *PAX3* slightly more than *MSX1* compared with expansion conditions (3 µM CHIR plus 0.5 µM PMA; Figure 2A). When the concentration of CHIR was reduced from 3 µM to 1.5 µM, *MSX1* and *PAX3* were downregulated by about the same amount (Figure 2A). When neural epithelial cells were cultured with PMA alone, *MSX1* and *PAX3* were down-regulated compared to expansion conditions. In addition, PMA alone resulted in increased the expression of *NKX6-1*, *NKX2-1*, *OLIG2*, and *FOXA2* compared to expansion conditions (Figure 2A). Of interest, we found that expression of the neural progenitor marker *SOX1* increased in cultures treated with only PMA compared to cultures grown under expansion conditions (Figure 2A). These results are consistent with the developmental roles of WNT and SHH signaling [12], [19].

To confirm the qRT-PCR results, we performed immunostaining on neural epithelial cells cultured for 6 days with PMA, CHIR, or both. PMA alone resulted in decreased PAX3 expression as well as increased expression of NKX6.1, NKX2.2, and FOXA2 compared to expansion conditions as well as CHIR alone (Figure 2B). In addition, more cells expressed NKX6.1, NKX2.2, and FOXA2 at 1 µM PMA compared to 0.5 µM PMA. In contrast, fewer PAX3-positive cells were observed at 1 µM PMA than at 0.5 µM PMA. There was increased SOX1-staining intensity in cells treated with only PMA compared to cells cultivated under expansion conditions (Figure 2B). More cells were SOX1-positive with PMA compared to cells treated with CHIR alone (Figure 2B). These data are consistent with the qRT-PCR results and suggest that cultures of neural epithelial cells can be directed to differentiate into ventral neural tube- and neural crest-derived lineages using CHIR and PMA, respectively. However, these differences may reflect different subpopulations within the culture. Experiments below discriminate between these alternatives.

Next, we sought to determine if expression of markers of rostrocaudal identity by cultures of neural epithelial cells could be modulated by developmental factors. Retinoids are produced *in vivo* by somites and specify spinal cord fate, which can be mimicked *in vitro* with all-trans retinoic acid (RA) [20]. Neural epithelial cells at passage 20 treated with RA for 1 or 8 days exhibited lower levels of *HOXA2* and increased levels of *HOXA4*, *HOXB4,* but not *HOXA9* (Figure 2C). Therefore, RA directs cultures of neural epithelial cells to express caudal patterning markers, consistent with the role of retinoids during embryonic development. In contrast, under none of the tested conditions were forebrain markers, such as *FOXG1*, induced, suggesting that neural epithelial cells are committed to expressing markers of caudal fates, consistent with the role of WNT factors in caudal fate specification.

### Directed Differentiation of Neural Epithelial Cells into Neural Crest Lineages: Peripheral Neurons and Mesenchymal Cells

**Figure 3:**
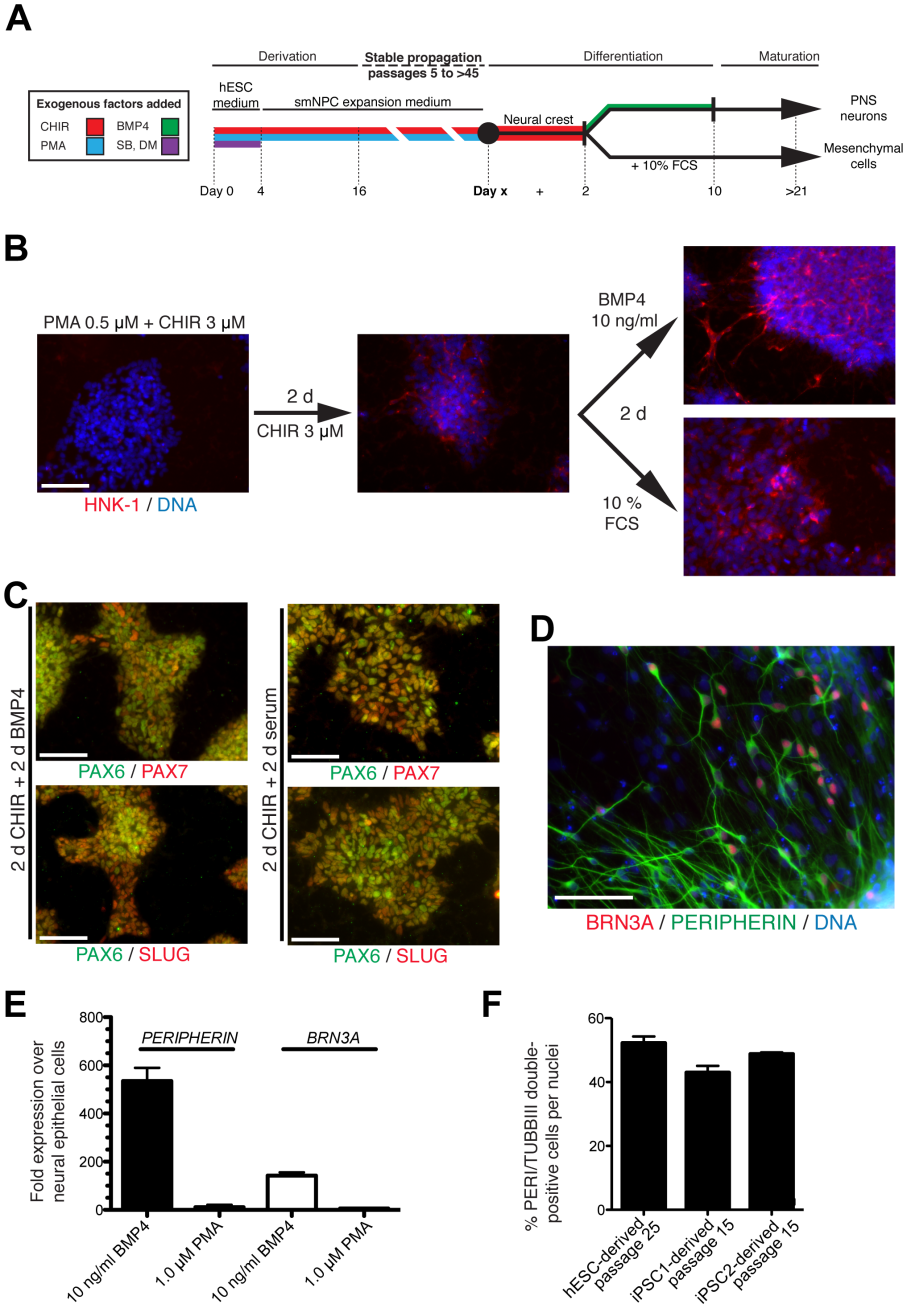
Differentiation of PNS neurons and mesenchymal cells from neural epithelial cells. (A) Summary of isolation and differentiation protocol used in this study. (B) Immunostaining of differentiated neural epithelial cells for HNK-1. (C) Neural epithelial cells were treated with CHIR for 2 days and then switched to BMP4 or serum-containing medium for 2 additional days. In both cases, the cultures show cells positive for the neural plate border/neural crest markers PAX7 and SLUG, whereas only some cells are still positive for PAX6. (D) Immunostaining of smNPCs differentiating in the presence of BMP4 for PERIPHERIN and BRN3A. (E) qRT-PCR demonstrating the upregulation of *PERIPHERIN* and *BRN3A* in neural epithelial cells differentiated for 8 days in the presence of BMP4, but not PMA, following two weeks of maturation. (F) More than 40% of cells are double positive for PERIPHERIN and TUBBIII after patterning with BMP4 and maturation. Error bars represent variation from 2 independent cultures. Scale bars are 100 µm. CHIR = CHIR99021, DM = dorsomorphin, FCS = fetal calf serum, PMA = purmorphamine, and SB = SB43152.

When cultured with only CHIR, cultures of neural epithelial cells expressed markers that might indicate potential to differentiate into neural crest cells. For this reason, we tested the ability of neural epithelial cells to differentiate into neural crest cells, by culturing cells at passages 15–25 in the presence of CHIR for 2 days followed by BMP4 without CHIR or 10% fetal calf serum (FCS; Figure 3A). After four days, cells exhibited upregulation of PAX7, SLUG, and the neural crest surface marker HNK-1, but downregulation of PAX6 (Figures 3B and C). qRT-PCR demonstrated that *PAX7*, *SOX9* and *TFAP2A* were induced by BMP4 (Figure S6A). Immunostaining showed that 2 days of CHIR and BMP4 induced expression of TFAP2A (Figure S6B). This combination of markers is consistent with neural crest progenitors.

After maturation for two weeks, we observed greater than 80% of neurons expressed PERIPHERIN (Figure 3D). Some of the PERIPHERIN-positive neurons expressed the neural crest marker TFAP2A (Figure S5C). Immunostaining and confocal microscopy demonstrated that a subset of cells expressed both PERIPHERIN and BRN3A, which is a combination that specifically marks PNS sensory neurons (Figure 3D and Figure S6D). After differentiation and maturation, qRT-PCR analyses confirmed that peripheral sensory neuron markers *PERIPHERIN* and *BRN3A* were upregulated by BMP4 (Figure 3E). As expected, 8 days of treatment with PMA instead of BMP4 essentially abolished expression of these markers (Figure 3F). Overall, the efficiency of directing differentiation of neural epithelial cells into PERIPHERIN and TUBBIII double-positive cells was about 40 to 50% (Figure 3F). We conclude that cultures of neural epithelial cells are capable of forming cells expressing markers of peripheral neurons, including sensory neurons.

Neural crest cells can form non-neural cells including mesenchymal cells. To test whether neural epithelial cells can also form non-neural cells, the cells at passages 20–25 were differentiated using first CHIR only for 2 days and then serum-containing medium for 14–21 days (Figure 3A). Under these conditions, we observed the formation of mesenchymal cells that could be cultured for more than 10 passages and resembled primary human fibroblasts. Human fibroblasts, which are mesenchymal cells, as well as the differentiated mesenchymal cells expressed the markers VIMENTIN, CD9, SMA, NESTIN, and alkaline phosphatase (Figure S6E). Expression of SMA and alkaline phosphatase may indicate spontaneous differentiation of cells into smooth muscle and osteoblastic cells. Finally, using mesenchymal stem cell protocols, we were able to differentiate the neural epithelial cell–derived mesenchymal cells into cells expressing markers of adipocytes and osteocytes (Figure S6F). Based on these data, we conclude that cultures of neural epithelial cells are capable of forming cells expressing markers of peripheral neurons as well as mesenchymal neural crest cell derivatives.

### Directed Differentiation of Neural Epithelial Cells into Ventral Neural Tube Lineages: mDANs and MNs

**Figure 4:**
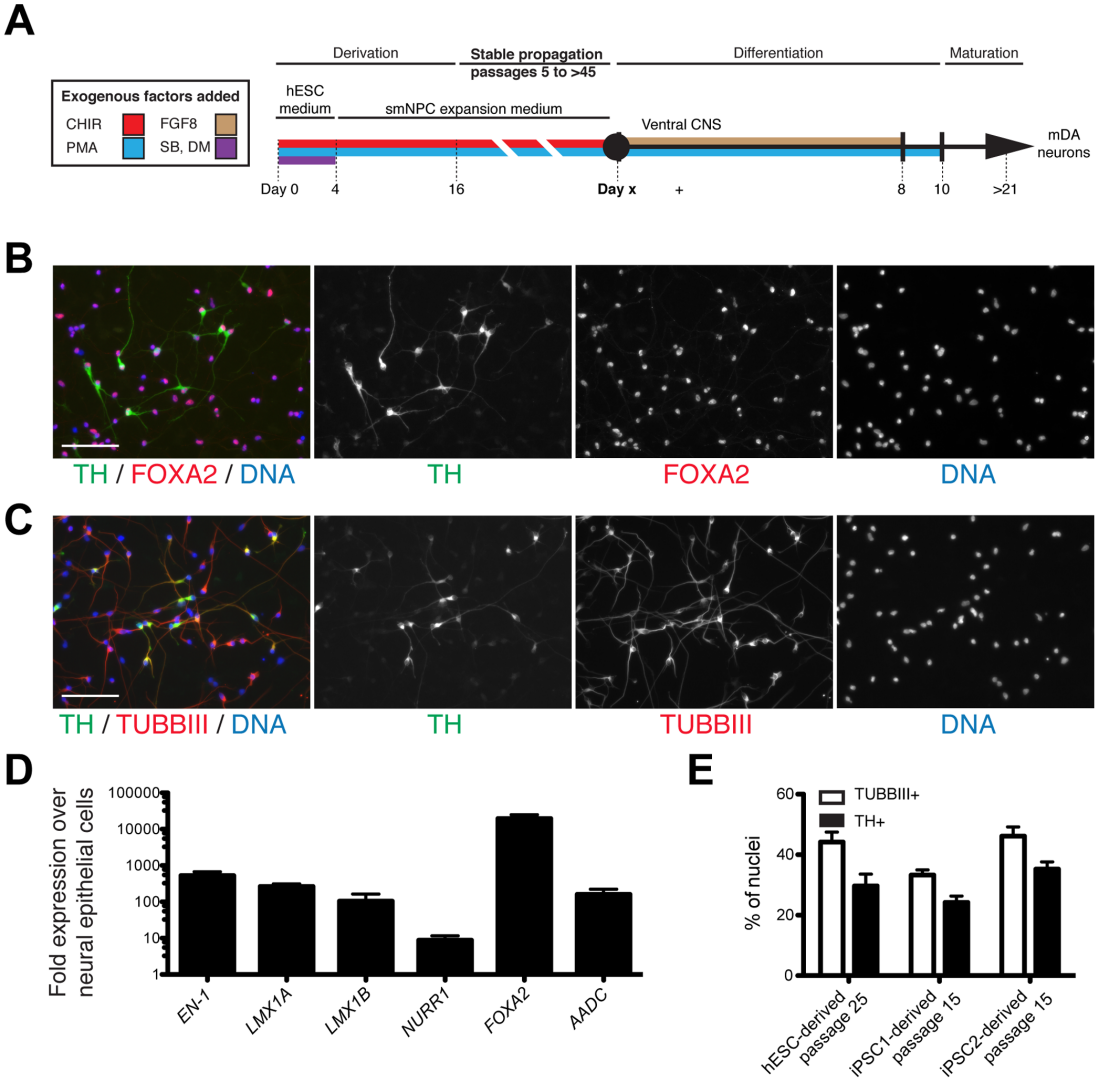
Directed differentiation of neural epithelial cells into mDANs. (A) Summary of isolation and differentiation protocol used in this study. (B) hESC-derived neural epithelial cells were differentiated into mDANs and immunostained for TH and FOXA2 and counterstained for nuclei with Hoechst. (C) Immunostaining of neural epithelial cell–derived mDANs for TH and TUBIII and counterstained for nuclei with Hoechst. (D) qRT-PCR analysis of neural epithelial cell–derived cultures for the indicated markers of mDAN specification on day 21. Error bars show standard deviation from 3 different experiments. (E) Efficiency of mDAN formation for 3 independent neural epithelial cell lines. Error bars represent the variation between 2 independent cultures. Scale bars are 100 µm. CHIR = CHIR99021, DM = dorsomorphin, PMA = purmorphamine, and SB = SB43152.

When cultured with only PMA, cultures of neural epithelial cells expressed markers that might indicate potential to differentiate into ventral neural tube cell lineages. For this reason, we tested the ability of neural epithelial cells to differentiate into mDANs and MNs, which are derived from ventral neural tube progenitors *in vivo*. First, we exposed neural epithelial cells at passages 15–25 to PMA and FGF8 for 8 days (Figure 4A), which specify formation of ventral midbrain cells including mDANs [21]. After maturation for 2 weeks, immunostaining of neural epithelial cells demonstrated that a large proportion had differentiated into TH-, FOXA2-, and TUBBIII-positive neurons, an expression pattern that specifically marks mDANs (Figures 4B and C). qRT-PCR showed upregulation of markers of mDAN differentiation, including *EN-1*, *LMX1A*, *LMX1B*, *NURR1*, *FOXA2*, and *AADC* (Figure 4D). The overall efficiency (up to ∼35% of total cells and up to 70% of neurons) of differentiation of neural epithelial cells into cells expressing markers of mDAN identity was consistent among 3 different neural epithelial cell cultures at multiple different passage numbers from three different pluripotent stem cell lines (Figure 4E). Thus, cultures of neural epithelial cells have the potential to differentiate into cells expressing markers indicative of mDAN identity.

**Figure 5:**
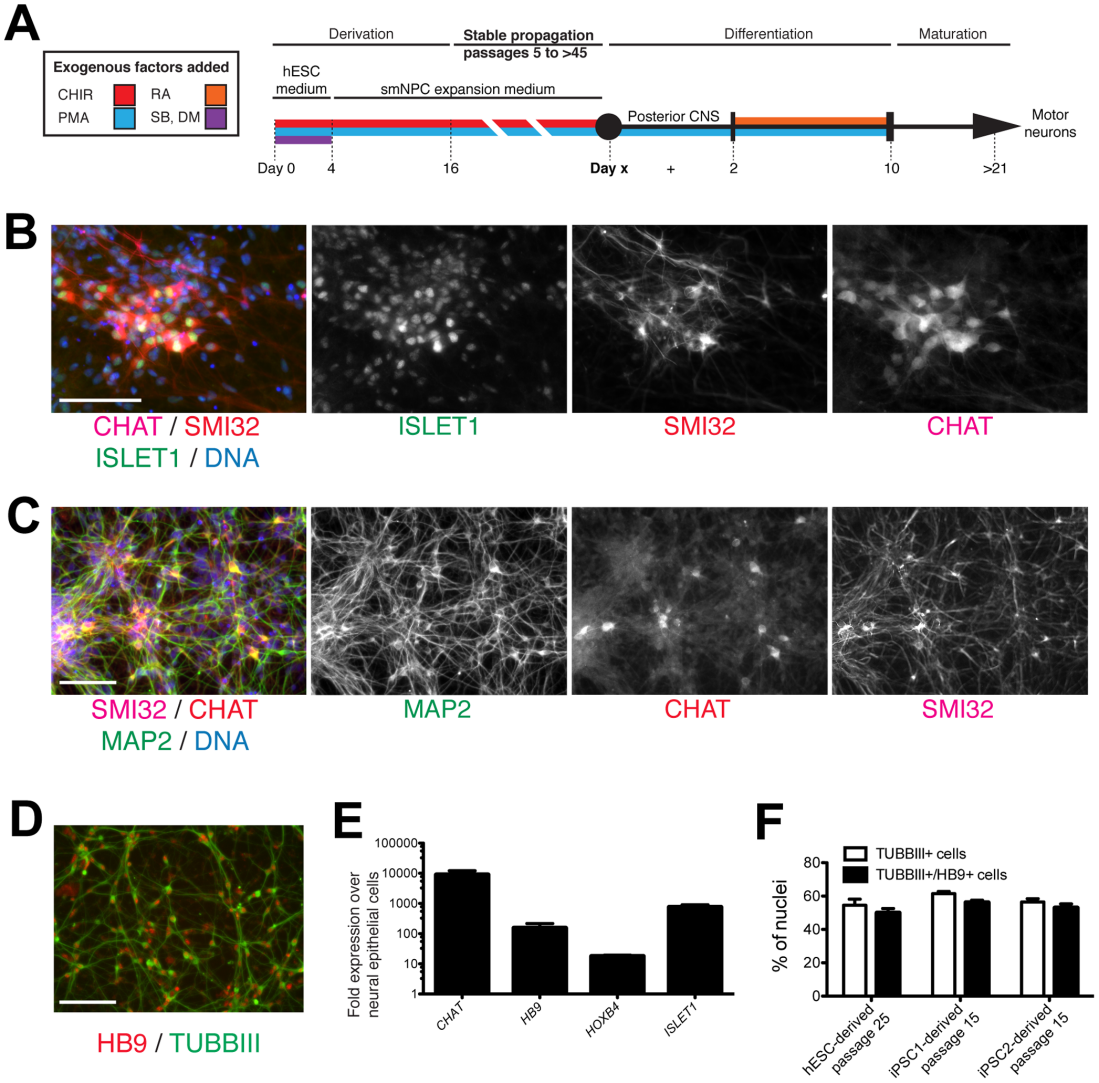
Directed differentiation of neural epithelial cells into MNs. (A) Summary of isolation and differentiation protocol used in this study. (B) hESC-derived neural epithelial cells were differentiated into MNs and immunostained for ISLET1, CHAT, and MAP2 and counterstained for nuclei with Hoechst. (C) Immunostaining showing colocalization of ISLET1, SMI32 and CHAT. (D) Immunostaining of neural epithelial cell–derived MNs showing colocalization of HB9 and TUBIII. (E) qRT-PCR analysis of neural epithelial cell–derived cultures for the indicated markers of MN specification on day 21 of differentiation. Error bars show standard deviation of 2 independent experiments. (F) MN differentiation efficiency from neural epithelial cells was approximately 50% as determined by TUBBIII and HB9 colocalization. Error bars represent variation from 3 independent cultures. Scale bars are 100µm. CHIR = CHIR99021, DM = dorsomorphin, PMA = purmorphamine, RA = all-trans retinoic acid, and SB = SB43152.

SHH and RA signaling in combination specify the formation of MNs [22]. As the expression of markers of neural fate by neural epithelial cells is modulated by both SHH and RA, we asked whether both factors together can direct differentiation along the MN lineage. Neural epithelial cells at passages 15–25 were treated for 2 days with 1 µM PMA and then for 8 days with 1 µM PMA and 1 µM RA to induce ventralization and caudalization, with subsequent maturation for 2 weeks without PMA or RA (Figure 5A). Immunostaining of the cells demonstrated the presence of ISLET1 and CHAT double-positive cells that also expressed MAP2 and SMI32, consistent with MN identity (Figures 5B and C). MN identity is supported by the presence of HB9 and TUBBIII double-positive cells (immunostaining, see Figure 5D). qRT-PCR analysis showed significant upregulation of markers of MN differentiation including *HB9, ISLET1, CHAT,* and *HOXB4* (qRT-PCR, see Figure 5E). Counting of single cells after maturation showed that neural epithelial cells formed cells expressing markers of MN identity with an efficiency of approximately 50% (Figure 5F).

### Cultures of Neural Epithelial Cells Contain Multipotent Stem Cells Capable of Forming both Neural Tube and Neural Crest Cell Lineages

Although these results indicate that cultures of neural epithelial cells have the potential to differentiate into both neural crest and neural tube cell derivates, it is possible that these derivatives arise from multiple subpopulations of cells in these cultures separately committed to either neural tube or neural crest cell fates. We therefore asked whether single neural epithelial cells are multipotent and capable of forming both neural tube and neural crest cell lineages. To answer this question, we generated 3 clonal neural epithelial cells lines from a single hESC-derived culture of neural epithelial cells (Figure S7A). These clonal lines expressed the neural progenitor makers NESTIN, SOX2, SOX1, and PAX6 (Figure S7B). Each of these three lines could be differentiated into mDANs, MNs, and PNS neurons (Figure S7C). Finally, we demonstrated that each of these three lines could be directed to differentiate into cells expressing markers of astrocytes and olidodendrocytes (Figure S7D). Therefore, we concluded that cultures of neural epithelial cells contain cells that self-renew and are competent to differentiate into cells expressing markers consistent with caudal derivatives of both neural tube and neural crest cells. Because of their differentiation capacity as well as their ability to self-renew with only small molecules, we termed these cells small molecule neural progenitor cells (smNPCs).

### Neurons Formed from smNPCs are Electrophysiologically Functional, Integrate and Mature *in vivo*

**Figure 6:**
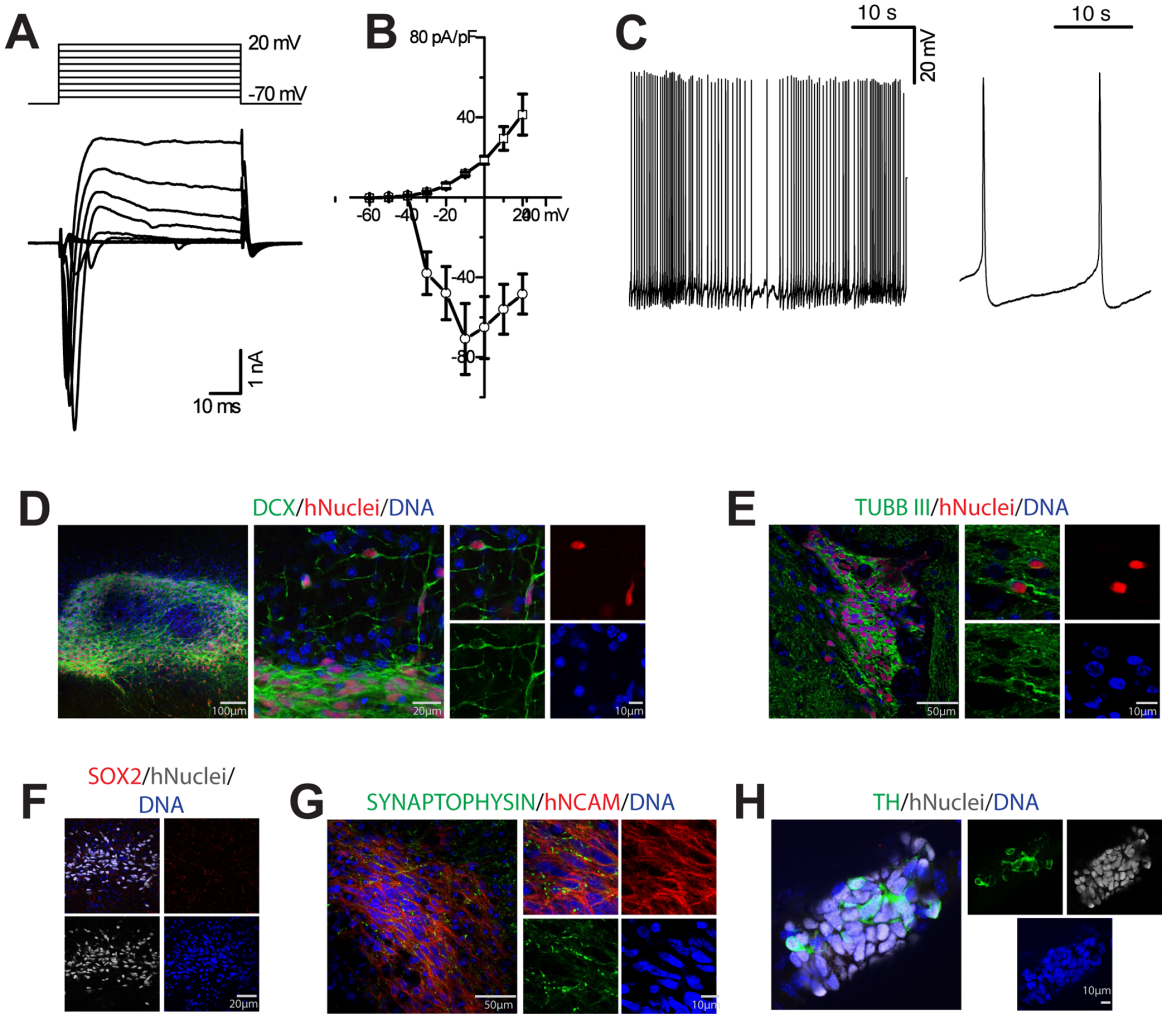
smNPCs-derived neurons become electrophysiologically mature and mature *in vivo.* (A) On average, the recorded membrane potential from smNPC-derived neurons was −35±2 mV (n = 12) and the cell membrane capacitance was 31.88±4.36 pF (n = 12). These values are consistent with previously published results of neurons differentiated from human stem cells [34], [35], [36]. The net of transmembrane currents, elicited by the voltage steps from holding potential −70 mV to +20 mV with 10 mV increments (the above panel shows the stimulation paradigm). (B) Current-voltage relationship of inward and outward currents, measured on the peak and normalized to cell capacitance (n = 8). (C) Cells demonstrate spontaneous firing of action potentials (APs) like neurons. Right panel shows more detailed view on the unitary action APs. See also Figure S8 for additional data and recording of miniature potentials. (D)–(H) smNPCs integrate and mature *in vivo*. Transplanted human cells were identified using human Nuclei (hNuclei)- and human NCAM (hNCAM)- specific antibodies. (D) Two weeks after transplantation into the midbrain of immunodeficient mice, smNPCs differentiated into DCX-positive and TUBBIII (E) neurons. (F) Two weeks post transplantation, smNPC are negative for SOX2. (G) smNPCs form synapses already two weeks post transplantation, as shown by SYNAPTOPHYSIN and hNCAM staining. (H) Only after treatment with PMA and FGF8 for 8 days before transplantation did TH+ neurons form. For further analysis and long-term survival after eight weeks, see Figure S9.

Our next objective was to evaluate the electrophysiological function of smNPC-derived mDANs using patch clamping after two weeks of maturation. Stepping the membrane holding potential from −70 to +20 mV with 10 mV increments elicited a fast-activating, fast-inactivating inward current followed by a slower activating, slowly deactivating outward current (Figure 6A). The I–V curves of both currents are typical for sodium inward and potassium outward currents through voltage-gated channels (Figure 6B) [23], [24]. Current-clamp recordings demonstrated the presence of neurons that spontaneously fired action potentials (APs) with frequencies of up to 2.1 Hz (mean 1.00±0.28 Hz, n = 12; Figure 6C). One critical test of neuronal identity is whether smNPC-derived neurons can form functional synaptic connections using spontaneous miniature events [25]. To this end, we used an in voltage clamp whole-cell configuration with a holding potential of −70 mV and with a frequency 0.35±0.11 Hz. The average amplitude of miniature spontaneous postsynaptic currents was 21.18±2.47 pA (peak value; n = 7 cells, 360 events analyzed). Representative trace and offline analysis results are shown in Figure S8A– F. The offline analysis revealed that recorded miniature spontaneous postsynaptic currents have the amplitude or kinetic parameters comparable to those of human neurons [26], [27], [28]. These results demonstrate that smNPC-derived neurons have acquired the electrical properties of excitable neurons and developed synaptic contacts between neurons.

Our final test was to evaluate the survival and differentiation potential of smNPCs *in vivo.* 1.5×10^5^ smNPCs and smNPCs differentiated with PMA and FGF8 for 8 days were stereotactically transplanted into the midbrain of adult mice. Two weeks and eight weeks after transplantation, transplanted cells were identifiable with species-specific antibodies against human Nuclei and human NCAM (Figure 6D–H and Figures S9A–F). *In vivo* neuronal differentiation of smNPCs was detectable by hNuclei/DCX double positive as well as hNuclei/TUBBIII double-positive immunostainings (Figures 6D and E and Figure S9B and C). Furthermore, the grafted cells differentiated and expressed mature neuronal markers indicated by the lack of SOX2 and hNuclei double-positive cells and the presence of hNCAM and SYNAPTOPHYSIN as well as of hNCAM and NeuN double-positive cells (Figures 6F, 6G and S9). Only when smNPCs were pre-differentiated with PMA and FGF8 and transplanted into the substantia nigra, did they continue to differentiate towards the dopaminergic subtype *in vivo*, as demonstrated by the presence of TH and FOXA2 double-positive cells (Figures 6H and S9F). Therefore, smNPCs are capable of differentiating into neurons, including dopaminergic neurons, *in vivo* after transplantation. smNPCs were also derived from iPSCs. iPSC-derived smNPCs expressed similar markers and had the same differentiation potential as hESC-derived smNPCs (Figure S10).

### smNPC-derived mDANs with LRRK2 G2019S are Susceptible to Degeneration

**Figure 7:**
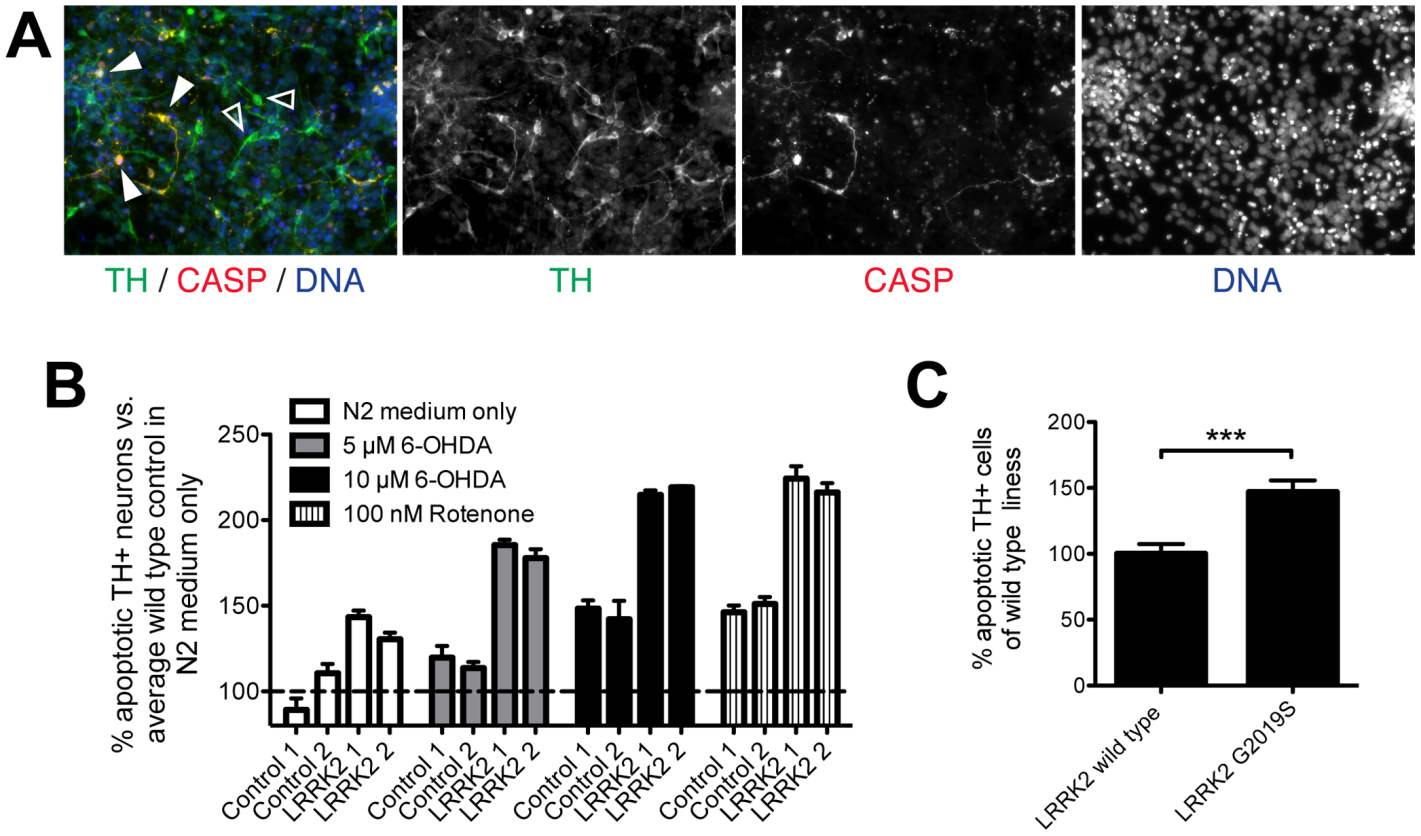
smNPCs are suitable for modeling of neurological diseases such as *LRRK2* G2019S induced Parkinson’s Disease. Patient-specific human iPSCs from two patients with Parkinson’s disease harboring *LRRK2* G2019S were differentiated in parallel with two lines derived from healthy age- and sex-matched control donors. After two weeks of maturation, the cultures were replated as single cells. Medium was switched to N2 medium or N2 medium supplemented with 5 µM 6-OHDA, or 10 µM 6-OHDA, or 100 nM Rotenone to induce additional cytotoxic stress. Apoptosis was assessed by double-staining for TH and cleaved CASPASE3 (CASP) in duplicate wells for each line and concentration. (A) Example picture showing TH+/CASP3- (empty arrowhead) and TH+/CASP3+ neurons (arrowhead). (B) When normalized to the average number of apoptotic cells detected in the wild-type cultures, 6-OHDA and rotenone lead to a higher cell death, with an even higher increase in cells carrying *LRRK2* G2019S. Error bars represent the variation from duplicate wells. (C) When normalizing each concentration to the average apoptosis in TH+ neurons from healthy controls, an increase of 46% can be observed in LRRK2 G2019S over wild type cultures in all stressor concentrations used. Error bars represent S.D. *** indicates p<0.001, according to Student’s t-test. See also Figure S11 for primary, unnormalized data.

Next, we assessed the ability of smNPCs to recapitulate neurodegeneration-associated pathology *in vitro*. For this experiment, smNPCs were derived from iPSCs generated from patients with PD carrying the mutation *LRRK2* G2019S as well as from age- and gender-matched controls. These smNPCs were then matured into cultures of mDANs for two weeks. First, we determined the affects of oxidative stress by culturing replated neurons in N2 medium alone to eliminate the antioxidants present in the B27 supplement and the neurotrophins used in the standard culture medium. After 48 hours, we observed an increase of 37% in cultures with *LRRK2* G2019S in the number of TH and cleaved CASPASE3 double-positive cells, which marks dopaminergic neurons undergoing apoptosis (Figure 7 and Figure S11). Addition of 6-hydroxydopamine or rotenone resulted in a significant increase in the number of TH and cleaved CASPASE3 double-positive cells (Figure 7 and Figure S11). These results are in agreement with the previously published phenotype [3]. Under all stressing conditions, more than 80% of the cells that were cleaved CASPASE3-positive also expressed TH (Figure S12). Therefore, apoptosis is preferentially induced in mDA neurons under the tested conditions. However, in contrast to the previous publication, this protocol is rapid, efficient, uses robustly expandable cells, and involves no manual manipulation. These characteristics should make smNPC-derived disease models more easily amenable to HTS compared to previous cell types.

## Discussion

**Figure 8:**
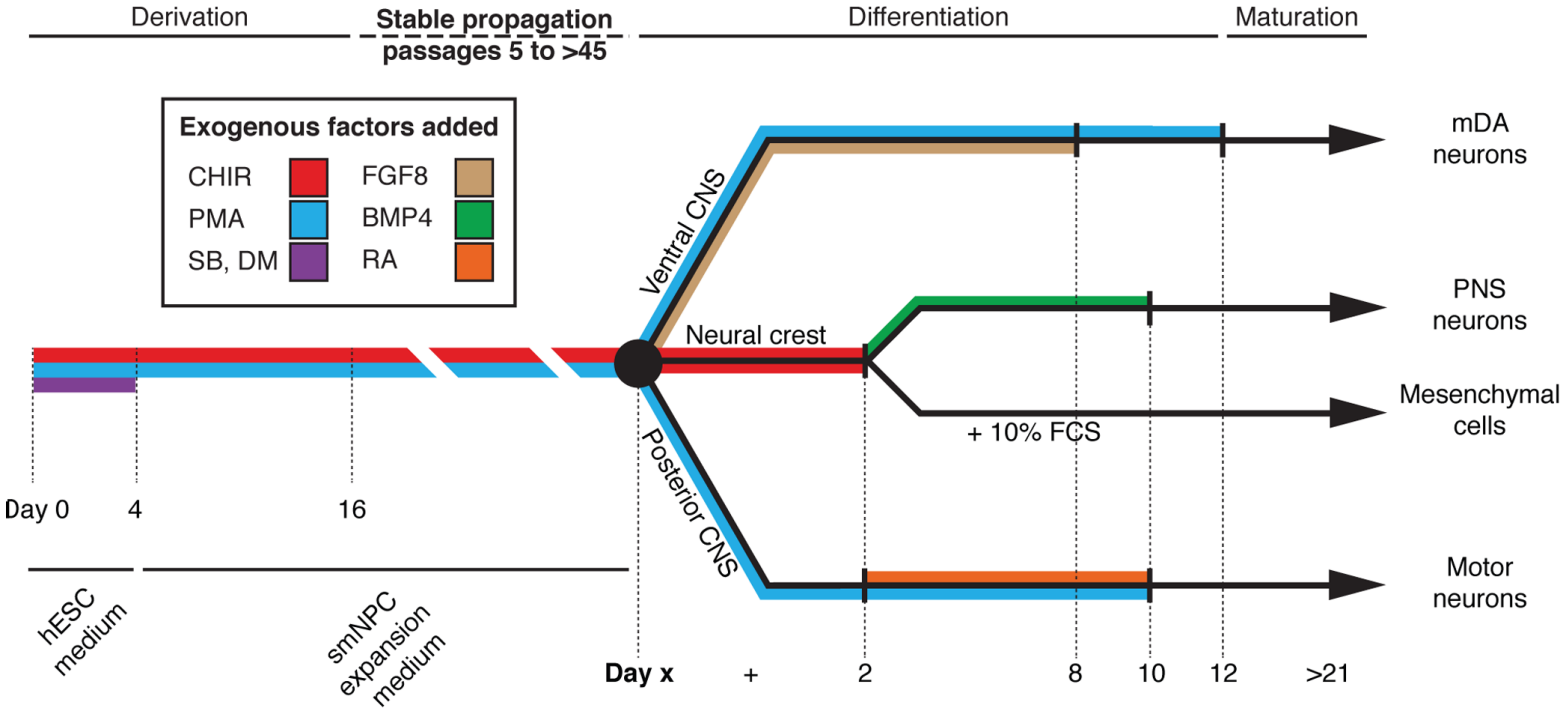
Summary of smNPCs. Diagram illustrating the conditions used to derive, propagate, and differentiate smNPCs. CHIR = 99021, DM = dorsomorphin, FCS = fetal calf serum, PMA = purmorphamine, RA = all-trans retinoic acid, and SB = SB43152.

HTS on stem cell-based phenotypic assays have the potential to discover revolutionary new drugs to treat neurodegenerative diseases. However, the scale of HTS campaigns requires a source of cells capable of robust and immortal expansion without costly growth factors or cumbersome manual steps. In addition, the cell source needs to be able to efficiently differentiate into lineages such as MNs and mDANs for disease modeling. Here, we have demonstrated that smNPCs possess these properties (Table 1 and Figure 8). No other reported cell type such as NSCs, lt-hESNSCs, pNSCs, R-NCs or even direct differentiation of hPSCs can meet these requirements. Although one report demonstrates the use of NSCs derived from hESCs for HTS, it is interesting to note that the screen was for small molecules that were selectively toxic to cells that were not dopaminergic neurons [29]. While this does, indeed, establish proof-of-principle for HTS using human NSCs, we argue that using inefficient differentiation protocols together with chemicals that are toxic to most of the resulting cells is likely to introduce many artifacts and is clearly not an optimal approach.

**Table 1:** Summary of the markers used in this study as well as the characteristics of NSCs, lt-hESNSCS, R-NCs, pNSCs, and smNPCs.

			Cultured Cell Type				
		Markers	NSCs	lt-hESNSCs	pNSCs	R-NCs	smNPCs
	**Origin**	Not applicable	Fetal/adult brain	hPSCs	hPSCs	hPSCs	hPSCs
	**Immortal self-renewal?**	Not applicable	Yes	Yes	Yes	No (very limitedexpansion)	Yes
	**Self-renewal with only small molecules?**	Not applicable	No	No	No	No	Yes
Differentiationpotential	**Neural crest**	PAX3, MSX1, SOX9, SLUG, PAX7, TFAP2A	No	Not tested	No	Yes	Yes
	**Peripheral neurons** (neural crest-derived)	PERIPHERIN, BRN3A, TFAP2A	No	Not tested	No	Yes	Yes
	**Mesenchymal cells** (neural crest-derivedlineage)	NESTIN, VIMENTIN, CD9	No	Not tested	No	Not tested	Yes
	**Osteoblasts** (mesenchymal cell-derived)	SMA	No	Not tested	No	Not tested	Yes
	**Osteocytes** (mesenchymal cell-derived)	OSTEOCALCIN, AP	No	Not tested	No	Not tested	Yes
	**Adipocytes** (mesenchymal cell-derived)	FABP4	No	Not tested	No	Not tested	Yes
	**Neural rosettes** (neural tube lineage)	ZO1, DACH1, PLZF, LMO3, NR2F1, PLAGL1, EVI1, LIX1	No	Not tested	Yes	Yes	
	**Neural progenitors** (neural tube lineage)	PAX6, SOX1, SOX2, NESTIN	Yes	Yes	Yes	Yes	Yes
	**Ventral neural tube** (neural tube-derivedlineage)	NKX6.1, NKX2.1, NKX2.2,OLIG2, FOXA2	Mixture	Yes	Yes	Yes	Yes
	**mDANs** (ventral neural tube-derived lineage)	TH, FOXA2, EN1, LMX1A, LMX1B, NURR1, AADC	No/very few	Yes (30%)	Yes (69–80%)	Yes	Yes (70%)
	**MNs** (ventral neural tube-derived lineage)	ISL1, HB9, CHAT, SMI32	No/very few	Yes (15%)	Yes (71%)	Yes (25%)	Yes (50%)
	**Neurons**	TUJ1, MAP2, NeuN, DCX, SYNAPTOPHYSIN	Yes	Yes	Yes	Yes	Yes
	**Astrocytes**	GFAP, S100-b	Yes	Yes	Yes	Yes	Yes
	**Oligodendrocytes**	O4, OLIG2	Yes	Yes	No	Yes	Yes

Differentiation efficiencies are reported in % of neurons and not of total cells.

The relationship of cell types derived *in vitro* such as lt-hESNSCs and pNSCs, with specific cell populations in developing embryos is of significant interest [13]. Unfortunately, such a comparison is not possible at present because data directly comparing the gene expression of specific embryonic cell populations with cells generated *in vitro* is lacking. Nevertheless, it is possible to speculate about the order of these cells based upon their reported differentiation potential. Pluripotent stem cells, of course, have the greatest differentiation potential of any cell type than can be cultured *in vitro*. Next, smNPCs and R-NCs have the competence to form both neural tube and neural crest lineages. In contrast, pNSCs are restricted to the CNS and unable to differentiate into PERIPHERIN-positive neurons [7]. However, pNSCs are able to efficiently form both mDANs and MNs. Finally, NSCs are restricted to the CNS and are unable to efficiently form mDANs and MNs. Although the differentiation potential of lt-hESNSCs for neural crest has not been robustly tested, treatment of lt-hESNSCs with BMP4 after pre-treatment with valproic acid and 5′-aza-2′-deoxycytidine resulted in only 5% of cells expressed PAX3 [30]. This strongly suggests that lt-hESNSCs are restricted to CNS fates. Like NSCs, they require FGF2 and EGF for self-renewal, and their ability to form mDANs and MNs is significantly reduced compared to pNSCs. For these reasons, we would order the cell types with decreasing differentiation potential as follows: hPSCs>smNPCs and R-NCs >pNSCs>lt-hESNSCs>NSCs.

## Materials and Methods

### Ethics Statement

Informed consent was obtained for all patients donating samples to this study prior to the donation using a written form and protocol that was prior approved by the instutional review board: Ethik-Kommission der Medizinischen Fakultät am Universitätsklinikum Tübingen. All experiments involving animals (e.g. cell transplantation) were carried out in accordance with local institutional guidelines under the protocol 87-51.04.2011.A057, which was approved by Landesamt für Natur, Umwelt und Verbraucherschutz of the state of North Rhine-Westphalia, Germany. In vitro experiments were carried out with existing cell lines obtained from previous studies except where noted below. The appropriate citations are given next to each cell line in the Materials and Methods.

### Generation of iPSCs

The iPSCs used in this study were newly generated. Informed consent was obtained from all patients involved in our study prior to cell donation as described in the ethics section above. Dermal fibroblasts, obtained from skin biopsies of patients with PD and healthy controls, were cultured in fibroblast medium, which consisted of DMEM supplemented with 10% fetal calf serum, 1% penicillin/streptomycin/glutamine, 1% nonessential amino acids, 1% sodium pyruvate (all PAA), and 0.5 mM beta-mercaptoethanol (Invitrogen).

The reprogramming of human dermal fibroblasts was adapted from Takahashi *et alia* [1]. Retroviral vectors containing *OCT4* (Addgene 17217), *SOX2* (Addgene 17218), *KLF4* (Addgene 17219), and, when indicated, *c-MYC* (Addgene 17220) were co-transfected using Fugene 6 (Roche) into 293 T cells (purchased from ATCC) together with the appropriate packaging plasmids (Addgene 8454 and 8449). After 48 hours, supernatants containing viral particles were applied to the patients’ fibroblasts in the presence of 6 mg/mL protamine sulphate (Sigma Aldrich). Two to four infections were performed for each fibroblast sample. One day later, fibroblasts were reseeded on mouse embryo fibroblast (MEF) feeder cells or on gelatin-coated cell culture dishes. The mouse embryonic fibroblasts (MEFs) used in this study were derived in the laboratory of Prof. Dr. Hans Schöler and have been reported previously [31]. The following day, hESC medium supplemented with 1 mM valproic acid (Sigma Aldrich) was added, and the culture medium was changed daily thereafter. After 10–14 days, iPSC-like colonies were observed and valproic acid was discontinued. Individual colonies were isolated and clonally expanded. In total, two iPSC lines, designated Control 1 and Control 2, were derived from healthy patients and two iPSC lines, designated LRRK2 1 and LRRK2 2, were derived from patients with PD harboring the mutation G2019S.

### Pluripotent Stem Cell Culture

The human ESC line HUES6 was used in this study and was purchased from the hESC Collection (Harvard University). The derivation of this line has been reported previously [32]. HUES6 and iPSCs were cultured on a layer of mitotically inactivated (with mitomycin C (Tocris)) mouse embryo fibroblasts (MEFs) in hESC medium. The mouse embryonic fibroblasts (MEFs) used in this study were derived in the laboratory of Prof. Dr. Hans Schöler and have been reported previously [31]. hESC medium consisted of Knockout DMEM (Invitrogen) with 20% Knockout Serum Replacement (Invitrogen), 1 mM beta-mercaptoethanol (Invitrogen), 1% nonessential amino acids (NEAA, Invitrogen), 1% penicillin/streptomycin/glutamine (PAA), freshly supplemented with 5 ng/mL FGF2 (Peprotech). Pluripotent stem cells were split 1∶5 to 1∶8 every 5–7 days. Colonies were mechanically disaggregated with 1 mg/mL collagenase IV (Invitrogen). 10 µM ROCK Inhibitor (Ascent Scientific) was added for 24 hours after splitting.

### smNPC Derivation

For generation of smNPCs from pluripotent stem cells, colonies were detached from the MEFs 3–4 days after splitting, using 2 mg/mL collagenase IV. Pieces of colonies were collected by sedimentation and resuspended in hESC medium (without FGF2) supplemented with 10 µM SB-431542 (Ascent Scientific), 1 µM dorsomorphin (Tocris) for neural induction, as well as 3 µM CHIR 99021 (Axon Medchem) and 0.5 µM PMA (Alexis), and cultured in petri dishes. Medium was replaced on day 2 by N2B27 medium supplemented with the same small molecule supplements. N2B27 medium consisted of DMEM-F12 (Invitrogen)/Neurobasal (Invitrogen) 50∶50 with 1∶200 N2 supplement (Invitrogen), 1∶100 B27 supplement lacking vitamin A (Invitrogen) with 1% penicillin/streptomycin/glutamine (PAA). On day 4, SB-431542 and dorsomorphin were withdrawn and 150 µM Ascorbic Acid (AA; Sigma) was added to the medium. On day 6, the EBs, which showed intensive neuroepithelial outgrowth, were triturated with a 1,000 µL pipette into smaller pieces and plated on Matrigel-coated (Matrigel, growth factor reduced, high concentration; BD Biosciences) 12-well plates at a density of about 10–15 per well in smNPC expansion medium (N2B27 with CHIR, PMA, and AA). For coating, Matrigel was diluted to a final dilution of 1∶100 in Knockout DMEM (Invitrogen) prior to coating 500 µL per well of a 12-ell plate overnight. Coated plates were wrapped with parafilm and kept in the fridge for up to 1 month. The first split was performed at a 1∶5 to 1∶10 ratio on days 2 to 4 after plating. All the remaining splitting ratios were at least 1∶10. The higher splitting ratios selected better for smNPC colonies and led to a high purity with fewer splits. After a maximum of 5 splits, cultures were virtually free of contaminating non-smNPCs.

### smNPC Culture

smNPC were cultured on Matrigel-coated 12-well (Nunc) cell-culture plates. smNPC expansion medium consisted of N2B27 freshly supplemented with CHIR, PMA, and AA, with a medium change every other day. Typically, cells were split 1∶10 to 1∶15 every 5 or 6 days. For splitting, cells were digested into single cells for about 15 minutes at 37°C with prewarmed accutase (PAA). Cells were diluted in DMEM (PAA) for centrifugation at 200×g for 5 minutes. The cell pellet was resuspended in fresh smNPC expansion medium and plated on Matrigel-coated cell culture dishes.

### Differentiation of smNPCs

All differentiation experiments were conducted with smNPCs of passage 13 and above. For undirected differentiation, including neurons, astrocytes and oligodendrocytes, it was sufficient to change smNPC expansion medium to N2B27 medium without supplements. For obtaining more homogenously plated cultures, cells were digested to single cells with Accutase after two weeks of differentiation, replated on fresh matrigel-coated plates and further differentiated for at least one week. For better survival, 50 µM dbcAMP (Sigma Aldrich) was added after replating.

For generation of more ventral CNS neurons, including mDANs, smNPC expansion medium was changed 2 days after splitting to N2B27 medium with 100 ng/mL FGF8 (Peprotech), 1 µM PMA, and 200 µM AA. After 8 days in this medium, maturation medium–N2B27 with 10 ng/mL BDNF (Peprotech), 10 ng/mL GDNF (Peprotech), 1 ng/mL TGF-b3 (Peprotech), 200 µM AA, and 500 µM dbcAMP–was used for the maturation of neurons. 0.5 µM PMA was added to this medium for 2 more days. One day after changing to maturation medium, the cultures were split at a 1∶3 ratio as small clumps, or single cells after Accutase treatment, or earlier when cultures became over-confluent. Cultures were analyzed after 2 weeks in maturation conditions unless otherwise indicated.

For induction of posterior cells, including MNs, smNPC expansion medium was changed to N2B27 with 1 µM PMA 3 days after splitting. Two days later, 1 µM retinoic acid (RA, Sigma) and 1 µM PMA were added for 8 days. Following one day in maturation medium (N2B27 with BDNF, GDNF, and dbcAMP), cultures were also split as clumps or single cells after Accutase treatment at a ratio of 1∶2 to 1∶3. Cells were cultured in maturation medium for 2 weeks.

For generation of PNS neurons, smNPCs 2 days after splitting were switched to N2B27 with only CHIR for 2 days. Afterward, 10 ng/mL BMP4 (R&D Systems) was added for 8 days. Splitting and maturation was performed as described for the generation of MNs.

For directed astrocyte differentiation, smNPCs were cultured with 10 ng/ml FGF2 and 10 ng/ml EGF (Peprotech) for 2 days and later switched to N2 medium with 4% FCS (PAA) supplemented with 10 ng/ml CNTF (Peprotech) for at least 2 weeks. Cultures were split using accutase when confluent and replated on fresh Matrigel-coated plates. After withdrawal of CNTF, cells were treated with 500 µM dbcAMP in N2 medium with 4% FCS for at least one week, or could be expanded for several weeks in 4% FCS containing N2 medium using 10 ng/ml EGF before being treated with dbcAMP. After dbcAMP treatment, cells were kept in 4% FCS in N2 for at least one more week.

For mesenchymal neural crest differentiation, smNPCs were cultured with CHIR only for 2 days after splitting and subsequently changed to DMEM (PAA) with 10% FCS and 1% penicillin/streptomycin/glutamine. Cultures were split at a 1∶3 ratio when confluent using trypsin (Invitrogen) and cultured on cell culture–treated plastic dishes. Mesenchymal cells derived from smNPCs were differentiated into osteocytes and adipocytes for 14 days, using the Human MSC Functional Identification Kit (R&D Systems). The supplied reagents were used according to the manufacturer's instructions.

### Cytotoxicity Experiments

For assessing sensitivity for cytotoxicity of wild type or LRRK2 G2019S mDANs, patient-specific iPSC – derived smNPCs at passage 15 were differentiated as mentioned above. All splitting procedures were performed as single cells using Accutase treatment. After 14 days of differentiation, mDAN cultures were digested to single cells using Accutase and reseeded in maturation medium on Matrigel-coated 48well or 96well plates (Nunc) at 70,000 or 35,000 cells per well. Two days later, medium was changed against N2 medium (DMEM/F12 with 1% N2 supplement and 1% penicillin/streptomycin/glutamine) for six hours to remove antioxidants and enzymes present in B27 supplement. Medium was changed against fresh N2 medium or N2 medium supplemented with 5 µM 6-OHDA, or 10 µM 6-OHDA (Tocris), or 100 nM Rotenone (Sigma). Two days later, cells were fixed and stained for TH and cleaved CASPASE3, as mentioned below.

### Transplantation

For analyzing the *in vivo* differentiation potential of smNPCs, smNPC and mDAN progenitors were transplanted into the midbrain of male NOD.CB17-*Prkdc^scid^*/NCrHsd mice (purchased from Harlan; 8 weeks, ∼25 g). The latter were differentiated towards mDANs for 8 days as described previously. Before transplantation, the cells were dissociated to single cells for about 15 minutes at 37°C with pre-warmed accutase and resuspended in medium at a density of 5×10^4^ cells per microliter. For stereotactical transplantation, animals were deeply anesthetized by intraperitoneal injection of 0.017 ml of 2.5% Avertin per gram of body weight and positioned into a stereotatic frame (David Kopf Instruments, model 940). Injection of 3 µl of the cell suspension was performed using a Hamilton 7005KH 5 µl syringe. Franklin & Paxinos mouse brain atlas was consulted for assessing the stereotactic coordinates of the midbrain in relation to bregma (anteroposterior: −3 mm, mediolateral: ±1,5 mm, dorsoventral: −4,4 mm below skull).

### Immunocytochemistry

For confocal microscopy, cells were plated on Matrigel-coated glass coverslips. Cultures were fixed for 20 minutes with 4% paraformaldehyde (Electron Microscopy Sciences) in PBS (Invitrogen) and washed twice with PBS. Permeabilization and blocking was done in one step using 0.1% Triton X-100 (Sigma Aldrich), 10% FCS, and 1% BSA in PBS for 45 minutes. Plates or coverslips were washed once with 0.1% BSA in PBS and the primary antibodies were applied overnight at 4°C in 1% BSA in PBS. The next day, following one washing step with 0.1% BSA in PBS, secondary antibodies were applied for one hour at room temperature in 1% BSA in PBS. Finally, cells were washed three times with 0.1% BSA in PBS-T (0.05% Tween-20), including a Hoechst counterstaining for nuclei in the second washing step. Cells were mounted in Vectashield mounting medium (Vector Labs) and imaged on a Zeiss PALM/Axiovert fluorescence microscope or a Zeiss LSM700 confocal microscope. If necessary, images were merged using ImageJ and Adobe Photoshop.

To determine the efficiency of differentiation into specific neurons, after 2 weeks in maturation medium, cells were disaggregated and seeded at a density of 5×10^4^ cells per well in maturation medium on Matrigel-coated 48-well plates. The next day, the cells were fixed and stained, as mentioned above. Cell counting and evaluation of the differentiation efficiency was performed using Cellomics ArrayScan high content imager with the supplied software. 25 fields were taken from each well with a 10X magnification, and the total number of cells was determined by counting the Hoechst-positive nuclei. Three independently differentiated cultures were evaluated for each iPSC line. MN cultures were counted manually from 5 randomly taken pictures at 10X magnification from each well.

The primary antibodies used in this study are mouse anti-NESTIN (1∶150, R&D), goat anti-SOX1 (1∶150, R&D), rabbit anti-PAX6 (1∶300, Covance), goat anti-SOX2 (1∶200, Santa Cruz), mouse anti-FOXA2 (1∶100, Santa Cruz), rabbit anti-TH (1∶500, Pel Freez), sheep anti-TH (1∶400, Pel Freez), mouse anti-TUBBIII (1∶1000, Covance), rabbit anti-TUBBIII (1∶2000, Covance), rabbit anti-MAP2 (1∶1000, Santa Cruz), mouse anti-O4 (1∶100, Millipore), rabbit anti-ISLET1 (1∶1500, Abcam), mouse anti-HB9 (5 µg/mL own preparation from DSHB hybridoma), goat anti-CHAT (1∶100, Millipore), mouse anti-BRN3A (1∶500, Santa Cruz), rabbit anti-PERIPHERIN (1∶200, Millipore), rabbit anti-GFAP (1∶1000, Dako), mouse anti GFAP (1∶500, Millipore), mouse anti S100-beta (1∶100, Thermo), mouse anti-SMA (1∶150, Dako), goat anti-T (1.200, Santa Cruz), mouse anti-Vimentin (1∶150, Dako), rabbit anti-CD9 (1.100, Santa Cruz), mouse anti-PAX7 (1∶100, Neuromics), mouse anti-SLUG (1∶100, Millipore), mouse anti-OSTEOCALCIN (R&D Systems, 5 µg/mL), goat anti-FABP4 (R&D Systems, 5 µg/mL) mouse anti-FORSE 1 (DSHB, 10 µg/mL), mouse anti-PAX3 (DSHB, 5 µg/mL), mouse anti-NKX2.2 (DSHB, 5 µg/mL), mouse anti-NKX6.1 (DSHB, 5 µg/mL), mouse anti TFAP2A (DSHB, 2 µg/mL), rabbit anti cleaved CASPASE3 (1∶1000, CellSignaling). All secondary antibodies were obtained from Invitrogen and were conjugated to AlexaFluor fluorochromes. Alkaline phosphatase activity was detected using FastRed and Naphtol (both Sigma) for 15 minutes at room temperature. Alkaline phosphatase-positive cells were recorded using the Texas Red fluorescence channel.

### Perfusion, Sectioning and Immunohistochemistry

Two and eight weeks after transplantation, anesthetized animals were intracardially perfused with 50 ml 1×PBS following 50 ml 4% PFA/1 PBS solution. The brains were isolated and post-fixed with 4% PFA/1 PBS solution over night at 4°C. A vibratome (Leica VT 1200 S) was used to prepare 40 µm sagittal sections. Permeabilization was performed by using TBS+/+/+ (TBS 0.1 M Tris, 150 mM NaCl, pH 7.4/0.5% Triton-X 100/0.1% Na-Azide/0.1% Na-Citrate/5% normal goat serum) for at least 1 h. Free floating sections were then incubated in TBS+/+/+ containing primary antibodies for 48 h on a shaker at 4°C, followed by 2 h incubation with Alexa-fluorophore conjugated secondary antibodies (Invitrogen) and Hoechst 33342 (Invitrogen) in TBS+/+/+ at room temperature. Following primary antibodies were used: DCX (1∶400, Abcam), TUBBIII (1∶600, Covance), human Nuclei (1∶200, Millipore), Synaptophysin (1∶200, Chemicon), NeuN (1∶400, Millipore), human NCAM (1∶100, Santa Cruz), FOXA2 (1∶100, Santa Cruz) and TH (1∶1000, Pel-Freez). Finally, sections were mounted in AquaMount (Dako) and analyzed with a Zeiss LSM 710 confocal microscope.

### Quantitative RT-PCR (qRT-PCR)

Total RNA was isolated from cultured cells using RNeasy columns (QIAGEN), according to manufacturer instructions, including an on-column DNase digest. Isolated RNA was reverse-transcribed using M-MLV Reverse Transcriptase (USB) with oligo-dT_16_ primers (Metabion) for 1 h at 42°C. qRT-PCR was performed on an Applied Biosystems 7500 Real-Time PCR system with SYBR green PCR master mix (ABI) and 56 ng of original RNA equivalents per 20 µL PCR reaction. Cycling conditions were 40 cycles of 15 s, 95°C/60 s 60°C. Relative expression levels were calculated using the 2^−2Δ^ method, normalized to biological reference samples and using *GAPDH* and *ACTB* as housekeeping genes.

### Whole Genome Expression Analysis

DNA-free total RNA samples (500 ng) to be hybridized on Illumina human-12 V3 expression BeadChips were processed using a linear amplification kit (Ambion) generating biotin-labeled cRNA (IVT duration: 14 h). This was quality-checked on a 2100 Bioanalyzer (Agilent) and hybridized as recommended and using materials and reagents provided by the manufacturer. In BeadStudio, raw data were background-subtracted and normalized using the “cubic spline” algorithm. Differential gene expression was assessed on the basis of thresholds for both expression ratios and statistical significance employing the “Illumina custom” algorithm considering standard deviations from replicate beads within each array. Signal intensities below 50% of the detection threshold were arbitrarily trimmed to the value corresponding to 50% of detection. This procedure underestimates expression changes for genes undetectable in the reference sample (or vice versa) but avoids nonsense ratios, such as negative or unrealistically high values.

### Karyotype Analysis

smNPCs at passage 25 were cultured until confluent. Three hours before chromosome preparation, colcimid (KaryoMAX; Invitrogen) was added to a final concentration of 0.3 µg/mL. After this incubation, the colcemid-containing solution was removed, the cells washed with PBS, and digested to a single-cell suspension with prewarmed Trypsin-EDTA, diluted in DMEM, and collected by centrifugation. The cell pellet was resuspended in 37°C prewarmed 75 mM KCl solution and incubated at room temperature for 7 minutes. The pellet was resuspended in ice-cold fixation solution (3∶1 methanol/acetic acid) while carefully shaking the cell suspension. Once fixed, the cells were collected by centrifugation and carefully resuspended in fresh fixative and incubated for 20 minutes at 4°C. Cells were spread by dropping different dilutions of cells in fixative on glass slides (Menzel Gläser, Thermo Scientific). The chromosomes were GTG-banded using standard procedures. Metaphase spreads were analyzed on a Zeiss AxioScop. 10 metaphases were analyzed from each line using the Cytovision software (Applied Imaging Corporation).

### Generation of Single-cell Clonal Lines

For the generation of single-cell clones, smNPCs were infected with a pLenti CMV -SV40-Blasticidine construct based on the pLenti6/V5 expression system (Invitrogen), which includes a blasticidin-resistance cassette. Virus production was performed in 293T cells using the ViraPower packaging mix (Invitrogen). One 6-cm plate of 293T cells were transfected using FuGENE 6 (Roche) according to the manufacturer’s instructions with 2 µg packaging mix and 1 µg expression construct. One day after transfection, medium was changed for N2B27 medium. The following day, the medium supernatant was filtered to remove 293T cells, supplemented with 6 µg/mL protamine sulfate (Sigma), 3 µM CHIR 99021, 0.5 µM PMA, 150 µM AA, and directly used for infection of freshly plated smNPC. The next day, infected smNPCs were washed 4 times with PBS and fed with fresh smNPC expansion medium. Selection with 5 µg/mL blasticidine (PAA) in smNPC expansion medium started 2 days later and was maintained for 2 more weeks.

Blasticidin-resistant smNPCs were digested and triturated to single cells using accutase for 30 minutes and filtered using a 40-µm cell strainer (BD Biosciences) to remove remaining cell aggregates. Single cells were counted and seeded at a density of 10 cells per well on a Matrigel-coated well of a 6-well plate, together with approximately 200,000 uninfected smNPCs in expansion medium. Four days later, cells were again selected with 5 µg/mL blasticidin, until only resistant, single colonies remained on the plate that were spotted and marked. Selection was maintained for 1 more week, single colonies were picked, replated on 4-well plates, and expanded under standard smNPC conditions, and blasticidin resistance was continued for 1 more week to exclude surviving non-resistant cells. Once sufficiently expanded, single cell–derived clones were differentiated as described above.

### Evaluation of Electrophysiological Function

The transmembrane current and spontaneous activity were recorded from smNPC-derived neurons, after 3 weeks of differentiation according to the mDAN protocol, using the whole-cell configuration of the patch-clamp technique [33]. The patch pipettes were fabricated from borosilicate glass on a PIP-6 pipette puller (HEKA Elektronik, Lambrecht, Germany). When filled with pipette solution, they had tip resistances of 5–7 MΩ. Recordings were done using a HEKA EPC-9 amplifier (HEKA Elektronik, Lambrecht, Germany) and Pulse 8.61 Aqusition Software (HEKA Elektronik, Lambrecht, Germany). Series resistance and pipette and whole-cell capacitance were cancelled electronically. Cells were perfused with a bath solution containing (mM): NaCl 140, KCl 2.4, MgCl_2_ 1.3, CaCl_2_ 2.5, HEPES 10, D-glucose 10, pH 7.4. The pipette solution contained (mM): K-gluconate 125, NaCl 10, EGTA 1, MgATP 4, HEPES 10, D-glucose 10, pH 7.4. All experiments were performed at room temperature. Recordings of current-voltage relationship (“I-V curves”) or miniature spontaneous activity (“minis”) were done in voltage-clamp mode at holding potential −70 mV. Recordings of spontaneous firing of action potentials (“AP”) were performed in current-clamp mode at 0 pA holding current, i.e. at own cell’s membrane potential.

Data were analyzed using Patcher’s Power Tool routine (developed by Dr. F. Mendez and F. Würriehausen, MPI BPC, Göttingen, Germany) for IgorPro (WaveMetrics, Lake Oswego, OR, USA) and Origin 7.5 (Origin Lab Corp., Northampton, MA, USA). Minis were analyzed with Mini Analysis 6.0 software (Synaptosoft Inc., Fort Lee, NJ, USA).

## Supporting Information

Figure S1 **Derivation of neural epithelial cells.** (A) Immunostaining of EBs with the indicted markers on day 6 of differentiation after being plated for 8 hours. (B) GTG-banded metaphase spreads for 3 independent neural epithelial cell lines derived from either human ESCs or iPSCs as indicated, analyzed at passage 25 to 27 and showing apparently normal diploid female karyotypes (46, XX). (C) Doubling time of neural epithelial cells derived from hESCs is stable over multiple passages. Neural epithelial cells from different pluripotent cell lines have comparable doubling times. 0.5 µM is the optimal PMA concentration for neural epithelial cell growth. When grown with PMA at a concentration of 0.25 µM or 1 µM, the doubling time was higher. Scale bars are 100 µm.
(TIF)

Figure S2 **smNPCs differentiate into oligodendrocytes after spontaneous differentiation.** (A) After at least three weeks of spontaneous differentiation by withdrawal of CHIR and PMA, single O4 positive cells are interspersed in the neural clusters, as identified by TUBBIII positive neurons. (B) Following reseeding as single cells and recovery of one more week, single O4/OLIG2 double positive oligodendrocytes can be identified. It is likely that this overall efficiency was diminished by the poor survival of oligodendrocytes to replating, which was necessary for the experiment.
(TIF)

Figure S3 **Gene expression by neural epithelial cells.** (A) Expression levels for the indicated genes derived from microarray analysis of 2 neural epithelial cell lines and their parental human pluripotent cell lines. Neural epithelial cells consistently expressed neural progenitor and rosette markers, but not markers of pluripotency or mesendodermal differentiation. (B) qRT-PCR analysis of 2 indicated neural epithelial cell lines at the indicated passage number for the indicated gene. *OCT4*, *SOX2* = pluripotency markers. *SOX2*, *SOX1*, *PAX6* = neural progenitor markers. *PAX3* = neural plate border marker. *FOXG1* = anterior neural progenitor marker. *AFP*, *SOX17* = endodermal markers. *T*, *CK8*, *CK18* = mesodermal markers. Microarray data has been deposited in NCBI under accession number GSE40556 and can be accessed through the link: http://www.ncbi.nlm.nih.gov/geo/query/acc.cgi?token=zzijjqwuasuugxe&acc=GSE40556
(TIF)

Figure S4 **Neural epithelial cells are capable of forming neural rosettes.** (A) Phase-contrast image of smNPCs before and after treatment with FGF2 for 2 days. (B) Immunostaining of neural epithelial cells before and after treatment with FGF2 for NESTIN, N-CADHERIN, and ZO-1. Arrowheads indicate N-CADHERIN and ZO-1 expression in the center of neural rosette-like structures, in contrast to the diffuse expression in neural epithelial cells. Scale bars are 100 µm.
(TIF)

Figure S5 **Neural epithelial cells express markers of a moderately dorsal, hindbrain character.** Microarray data of the indicated neural epithelial cell lines for markers of dorsoventral (A) and rostrocaudal (B) patterning. A schematic representation of the expression patterns of these markers *in vivo* in the developing neural tube is shown to the right. The black box indicates the approximate position of neural epithelial cells. See also Figure S2.
(TIF)

Figure S6 **Differentiation of neural epithelial cells into neural crest progenitors.** (A) qRT-PCR of neural epithelial cells after treatment with CHIR or BMP4 for 6 days for the neural crest markers *PAX7* and *SOX9* and *TFAP2A*. (B) Immunostaining for TFAP2A after differentiation of neural epithelial cells with CHIR for 2 days followed by BMP4 for two days, indicating a strong increase in TFAP2A positive cells. (C) After maturation, PNS-differentiated cells give rise to PERIPHERIN/TFAP2A double – positive cells. (C) Confocal imaging demonstrating BRN3A and PERIPHERIN double-positive peripheral neurons. (D) Immunostaining of primary human fibroblasts (hFib) or neural epithelial cell–derived mesenchymal cells differentiating and cultured with serum for the indicated mesenchymal markers. (E) After 14 days of differentiation with the Human Mesenchymal Stem Cell Functional Identification Kit (R&D Systems), neural epithelial cell–derived cells were assessed for alkaline phosphatase activity and OSTEOCALCIN expression, which are markers of osteocytes, as well as for cells containing oil droplets with FABP4 expression, which is a marker of adipocytes. Scale bars are 100 µm.
(TIF)

Figure S7 **Clonal differentiation into CNS and PNS lineages.** (A) Phase-contrast images of 3 independent single cell–derived neural epithelial cell colonies that were picked and expanded. (B) Immunostaining of the resulting clonal neural epithelial cell lines for the indicated neural progenitor markers. (C) Immunostaining for mDANs (TH/FOXA2), MNs (ISLET1/TUBIII), and PNS sensory (PERIPHERIN/BRN3A) neurons from the 3 clonal neural epithelial cell lines. (D) Clonally derived lines are multipotent and can give rise to neurons (MAP2), astrocytes (GFAP) and oligodendrocytes (O4/OLIG2) by spontaneous differentiation. Scale bars are 100 µm.
(TIF)

Figure S8 **smNPC-derived neurons acquire excitable properties of neuronal cells.** (A–E) Amplitude and kinetic parameters (n = 7 cells) and an exemplary recording of minis (F) performed at holding potential −70 mV after two to three weeks of maturation.
(TIF)

Figure S9 ***In vivo*** **differentiation potential and long-term survival of transplanted smNPCs.** (A) Already after two weaks of maturation *in vivo*, smNPCs form mature, postmitoctic neurons, as shown by NeuN and human – specific NCAM staining. (B–E) Eight weeks post transplantation, smNPCs have formed and survived as neurons and astrocytes, as shown by staining for DCX, TUBBIII and GFAP and form multiple synapses, as shown by staining for SYNAPTOPHYSIN. (F) After eight weeks, smNPCs prepatterned to an mDAN fate give rise to TH and FOXA2 double positive cells. Without prepatterning, cells are negative for FOXA2 (not shown). (G) Also mDAN-prepatterned smNPCs form synapses as shown by SYNAPTOPHYSIN staining.
(TIF)

Figure S10 **Human iPSC-derived smNPCs.** 2 independent smNPC lines were derived from iPSCs. The results of all experiments with hiPSC-derived smNPCs were directly comparable to those with hESC-derived smNPCs. (A) Immunostaining results for the indicated neural progenitor markers. Immunostaining of PNS neurons (B), mDANs (C), and MNs (D) differentiated from the iPSC-derived smNPCs. Scale bars are 100 µm.
(TIF)

Figure S11 ***LRRK2*** **G2019S increases sensitivity of dopaminergic neurons derived from smNPCs to stress compared with controls.** smNPCs were derived from two patient-specific LRRK2 mutant iPSCs, alongside with two age- and sex-matched controls. smNPCs were differentiated into mDANs, replated as single cells and incubated with N2 medium only, or supplemented with 5 µM 6-Hydroxydopamine (6-OHDA), or 10 µM 6-OHDA, or 100 nM rotenone. After two days, apoptotic mDANs were identified by immunostaining for TH and cleaved CASPASE3 (CASP3). Error bars represent variation between two independently stressed wells. (A) LRRK2 mutant mDANs show a higher degree of apoptosis in TH+ cells, as compared to healthy controls. (B) Additional stressors separate the mDAN cytotoxicity phenotype better between LRRK2 mutant and wild-type neurons than withdrawal of antioxidants and neurotrophins alone. *indicates p<0.05 according to the Student’s t-test.
(JPG)

Figure S12 **smNPC-derived mDANs are specifically susceptible to oxidative stress.** (A) Overview image of a stressed dopaminergic neuron culture after differentiation from smNPCs stressed with 6 OH-Dopa for two days before fixation. Cultures were stained with the indicated markers. Note that most of the cells positive for cleaved CASPASE3 (CASP3) as an indicator for apoptosis are also positive for TH. Only few CASP3+ cells are negative for TH (some indicated by arrowheads). The scale bar indicates 100 µm. (B) Stressed cultures were stained for TH and CASP3 and counted. The graph indicates a high specificity of stress-induced apoptosis for dopaminergic neurons as shown by CASP3 and TH double-positive cells. Under the tested conditions, more than about 80% of the CASP3-positive cells also express TH. The experiment was performed in duplicates. Error bars indicate variance.
(TIF)

Table S1 **Primers used for qRT-PCR used in this study.**
(DOCX)

## References

[1] Takahashi K, Tanabe K, Ohnuki M, Narita M, Ichisaka T, et al (2007) Induction of pluripotent stem cells from adult human fibroblasts by defined factors. Cell 131: 861–872. 18035408 10.1016/j.cell.2007.11.019

[2] Sanchez-Danes A, Richaud-Patin Y, Carballo-Carbajal I, Jimenez-Delgado S, Caig C, et al (2012) Disease-specific phenotypes in dopamine neurons from human iPS-based models of genetic and sporadic Parkinson's disease. EMBO Mol Med 4: 380–395. 22407749 10.1002/emmm.201200215 PMC3403296

[3] Nguyen HN, Byers B, Cord B, Shcheglovitov A, Byrne J, et al (2011) LRRK2 Mutant iPSC-Derived DA Neurons Demonstrate Increased Susceptibility to Oxidative Stress. Cell Stem Cell 8: 267–280. 21362567 10.1016/j.stem.2011.01.013 PMC3578553

[4] Karumbayaram S, Kelly TK, Paucar AA, Roe AJ, Umbach JA, et al (2009) Human embryonic stem cell-derived motor neurons expressing SOD1 mutants exhibit typical signs of motor neuron degeneration linked to ALS. Dis Model Mech 2: 189–195. 19259395 10.1242/dmm.002113 PMC2650191

[5] Conti L, Pollard SM, Gorba T, Reitano E, Toselli M, et al (2005) Niche-independent symmetrical self-renewal of a mammalian tissue stem cell. PLoS Biol 3: e283. 16086633 10.1371/journal.pbio.0030283 PMC1184591

[6] Koch P, Opitz T, Steinbeck JA, Ladewig J, Brustle O (2009) A rosette-type, self-renewing human ES cell-derived neural stem cell with potential for in vitro instruction and synaptic integration. Proc Natl Acad Sci U S A 106: 3225–3230. 19218428 10.1073/pnas.0808387106 PMC2651316

[7] Li W, Sun W, Zhang Y, Wei W, Ambasudhan R, et al (2011) Rapid induction and long-term self-renewal of primitive neural precursors from human embryonic stem cells by small molecule inhibitors. Proc Natl Acad Sci U S A 108: 8299–8304. 21525408 10.1073/pnas.1014041108 PMC3100988

[8] Elkabetz Y, Panagiotakos G, Al Shamy G, Socci ND, Tabar V, et al (2008) Human ES cell-derived neural rosettes reveal a functionally distinct early neural stem cell stage. Genes Dev 22: 152–165. 18198334 10.1101/gad.1616208 PMC2192751

[9] Gage FH (2000) Mammalian neural stem cells. Science 287: 1433–1438. 10688783 10.1126/science.287.5457.1433

[10] Patthey C, Gunhaga L, Edlund T (2008) Early development of the central and peripheral nervous systems is coordinated by Wnt and BMP signals. PLoS One 3: e1625. 18286182 10.1371/journal.pone.0001625 PMC2229838

[11] Patthey C, Edlund T, Gunhaga L (2009) Wnt-regulated temporal control of BMP exposure directs the choice between neural plate border and epidermal fate. Development 136: 73–83. 19060333 10.1242/dev.025890

[12] Lee KJ, Jessell TM (1999) The specification of dorsal cell fates in the vertebrate central nervous system. Annu Rev Neurosci 22: 261–294. 10202540 10.1146/annurev.neuro.22.1.261

[13] Conti L, Cattaneo E (2010) Neural stem cell systems: physiological players or in vitro entities? Nat Rev Neurosci 11: 176–187. 20107441 10.1038/nrn2761

[14] Kim DS, Lee JS, Leem JW, Huh YJ, Kim JY, et al (2010) Robust enhancement of neural differentiation from human ES and iPS cells regardless of their innate difference in differentiation propensity. Stem Cell Rev 6: 270–281. 20376579 10.1007/s12015-010-9138-1

[15] Chambers SM, Fasano CA, Papapetrou EP, Tomishima M, Sadelain M, et al (2009) Highly efficient neural conversion of human ES and iPS cells by dual inhibition of SMAD signaling. Nat Biotechnol 27: 275–280. 19252484 10.1038/nbt.1529 PMC2756723

[16] Zhang SC, Wernig M, Duncan ID, Brustle O, Thomson JA (2001) In vitro differentiation of transplantable neural precursors from human embryonic stem cells. Nat Biotechnol 19: 1129–1133. 11731781 10.1038/nbt1201-1129

[17] Bosse A, Zulch A, Becker MB, Torres M, Gomez-Skarmeta JL, et al (1997) Identification of the vertebrate Iroquois homeobox gene family with overlapping expression during early development of the nervous system. Mech Dev 69: 169–181. 9486539 10.1016/s0925-4773(97)00165-2

[18] Kiecker C, Niehrs C (2001) A morphogen gradient of Wnt/beta-catenin signalling regulates anteroposterior neural patterning in Xenopus. Development 128: 4189–4201. 11684656 10.1242/dev.128.21.4189

[19] Bang AG, Papalopulu N, Goulding MD, Kintner C (1999) Expression of Pax-3 in the lateral neural plate is dependent on a Wnt-mediated signal from posterior nonaxial mesoderm. Dev Biol 212: 366–380. 10433827 10.1006/dbio.1999.9319

[20] Novitch BG, Wichterle H, Jessell TM, Sockanathan S (2003) A requirement for retinoic acid-mediated transcriptional activation in ventral neural patterning and motor neuron specification. Neuron 40: 81–95. 14527435 10.1016/j.neuron.2003.08.006

[21] Gale E, Li M (2008) Midbrain dopaminergic neuron fate specification: Of mice and embryonic stem cells. Mol Brain 1: 8. 18826576 10.1186/1756-6606-1-8 PMC2569927

[22] Wichterle H, Lieberam I, Porter JA, Jessell TM (2002) Directed differentiation of embryonic stem cells into motor neurons. Cell 110: 385–397. 12176325 10.1016/s0092-8674(02)00835-8

[23] Simard JM, Song Y, Tewari K, Dunn S, Werrbach-Perez K, et al (1993) Ionic channel currents in cultured neurons from human cortex. J Neurosci Res 34: 170–178. 7680726 10.1002/jnr.490340204

[24] Cummins TR, Xia Y, Haddad GG (1994) Functional properties of rat and human neocortical voltage-sensitive sodium currents. J Neurophysiol 71: 1052–1064. 8201401 10.1152/jn.1994.71.3.1052

[25] Del Castillo J, Katz B (1954) Quantal components of the end-plate potential. J Physiol 124: 560–573. 13175199 10.1113/jphysiol.1954.sp005129 PMC1366292

[26] Inenaga K, Honda E, Hirakawa T, Nakamura S, Yamashita H (1998) Glutamatergic synaptic inputs to mouse supraoptic neurons in calcium-free medium in vitro. J Neuroendocrinol 10: 1–7. 9510053 10.1046/j.1365-2826.1998.00662.x

[27] Edwards FA, Konnerth A, Sakmann B (1990) Quantal analysis of inhibitory synaptic transmission in the dentate gyrus of rat hippocampal slices: a patch-clamp study. J Physiol 430: 213–249. 1707966 10.1113/jphysiol.1990.sp018289 PMC1181735

[28] Wyllie DJ, Manabe T, Nicoll RA (1994) A rise in postsynaptic Ca2+ potentiates miniature excitatory postsynaptic currents and AMPA responses in hippocampal neurons. Neuron 12: 127–138. 7507335 10.1016/0896-6273(94)90158-9

[29] Han Y, Miller A, Mangada J, Liu Y, Swistowski A, et al (2009) Identification by automated screening of a small molecule that selectively eliminates neural stem cells derived from hESCs but not dopamine neurons. PLoS One 4: e7155. 19774075 10.1371/journal.pone.0007155 PMC2743191

[30] Driehaus JV (2008) Regionalisation of human ES cell derived neural precursors. Bonn: Rheinischen Friedrich-Wilhelms-Universität Bonn. 124 p.

[31] Sterneckert J, Stehling M, Bernemann C, Arauzo-Bravo MJ, Greber B, et al (2010) Neural induction intermediates exhibit distinct roles of Fgf signaling. Stem Cells 28: 1772–1781. 20715182 10.1002/stem.498

[32] Cowan CA, Klimanskaya I, McMahon J, Atienza J, Witmyer J, et al (2004) Derivation of embryonic stem-cell lines from human blastocysts. N Engl J Med 350: 1353–1356. 14999088 10.1056/NEJMsr040330

[33] Hamill OP, Marty A, Neher E, Sakmann B, Sigworth FJ (1981) Improved patch-clamp techniques for high-resolution current recording from cells and cell-free membrane patches. Pflugers Arch 391: 85–100. 6270629 10.1007/BF00656997

[34] Westerlund U, Moe MC, Varghese M, Berg-Johnsen J, Ohlsson M, et al (2003) Stem cells from the adult human brain develop into functional neurons in culture. Exp Cell Res 289: 378–383. 14499639 10.1016/s0014-4827(03)00291-x

[35] Moe MC, Varghese M, Danilov AI, Westerlund U, Ramm-Pettersen J, et al (2005) Multipotent progenitor cells from the adult human brain: neurophysiological differentiation to mature neurons. Brain 128: 2189–2199. 15958504 10.1093/brain/awh574

[36] Coyne L, Shan M, Przyborski SA, Hirakawa R, Halliwell RF (2011) Neuropharmacological properties of neurons derived from human stem cells. Neurochem Int 59: 404–412. 21315124 10.1016/j.neuint.2011.01.022

