# Correction: Derivation and Expansion Using Only Small Molecules of Human Neural Progenitors for Neurodegenerative Disease Modeling

**DOI:** 10.1371/annotation/6a917a2e-df4a-4ad9-99bb-6aa7218b833e

**Published:** 2013-11-14

**Authors:** Peter Reinhardt, Michael Glatza, Kathrin Hemmer, Yaroslav Tsytsyura, Cora S. Thiel, Susanne Höing, Sören Moritz, Juan A. Parga, Lydia Wagner, Jan M. Bruder, Guangming Wu, Benjamin Schmid, Albrecht Röpke, Jürgen Klingauf, Jens C. Schwamborn, Thomas Gasser, Hans R. Schöler, Jared Sterneckert

The files for Figures S6 and S7 are incorrect. Please view the correct Figure S6 here: 

and view the correct Figure S7 here: 
