# Supplementary figures and images for: Derivation and Expansion Using Only Small Molecules of Human Neural Progenitors for Neurodegenerative Disease Modeling
Source: PLoS One. 2013 Mar 22;8(3):e59252. doi: 10.1371/journal.pone.0059252 (PMC3606479; doi:10.1371/journal.pone.0059252)

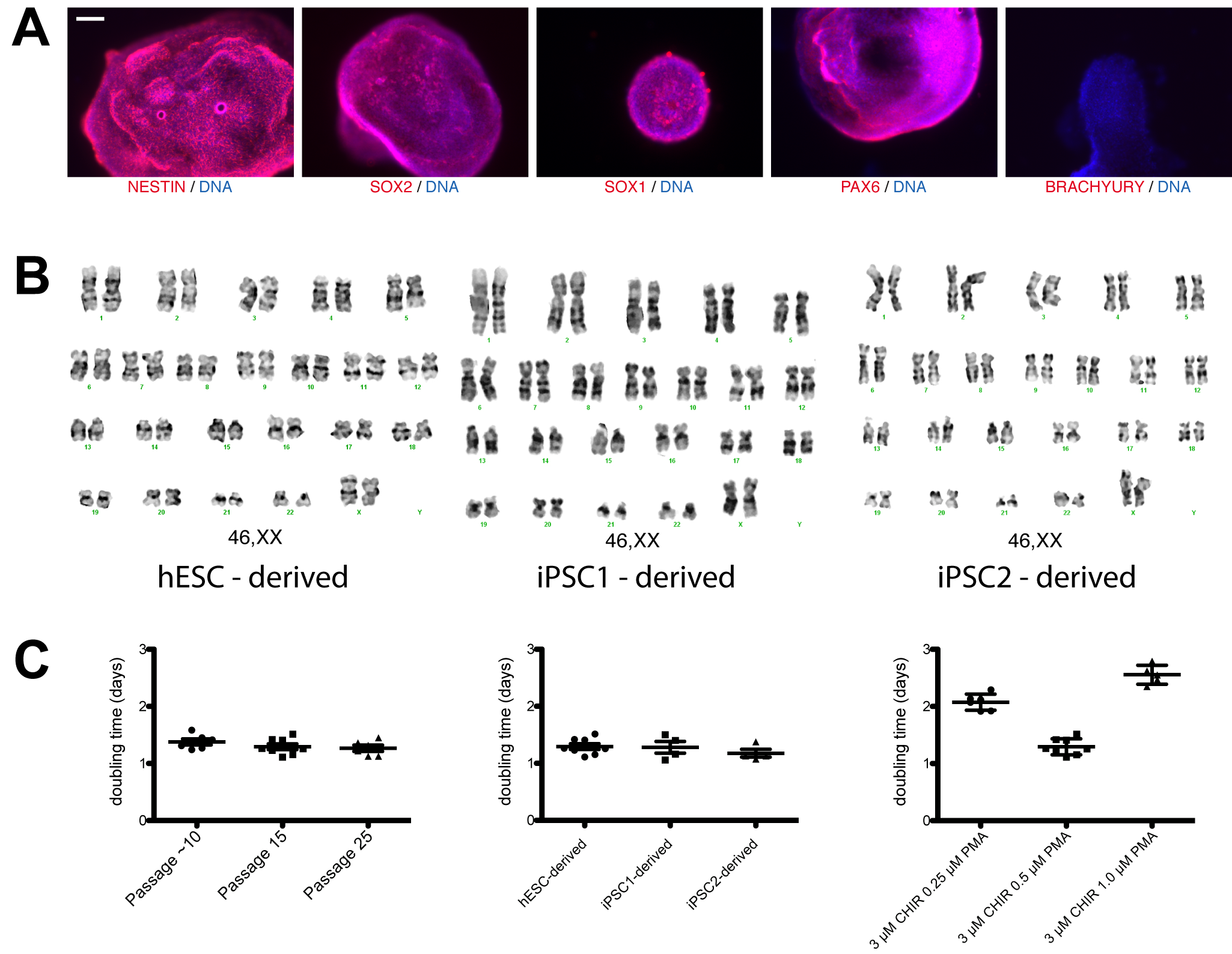

Supplement: Figure S1 — Derivation of neural epithelial cells. (A) Immunostaining of EBs with the indicted markers on day 6 of differentiation after being plated for 8 hours. (B) GTG-banded metaphase spreads for 3 independent neural epithelial cell lines derived from either human ESCs or iPSCs as indicated, analyzed at passage 25 to 27 and showing apparently normal diploid female karyotypes (46, XX). (C) Doubling time of neural epithelial cells derived from hESCs is stable over multiple passages. Neural epithelial cells from different pluripotent cell lines have comparable doubling times. 0.5 µM is the optimal PMA concentration for neural epithelial cell growth. When grown with PMA at a concentration of 0.25 µM or 1 µM, the doubling time was higher. Scale bars are 100 µm. (TIF) [file pone.0059252.s001.tif]

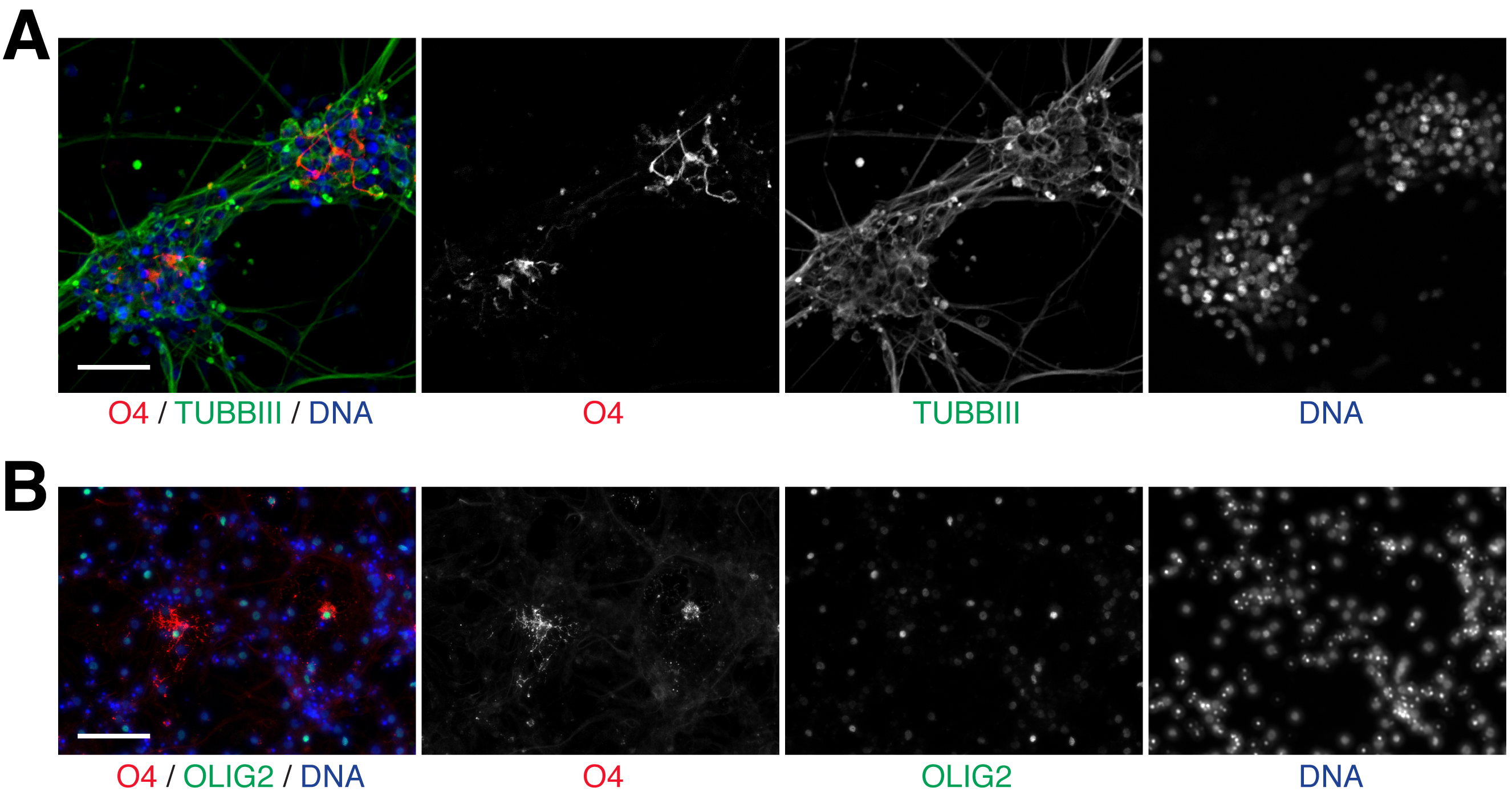

Supplement: Figure S2 — smNPCs differentiate into oligodendrocytes after spontaneous differentiation. (A) After at least three weeks of spontaneous differentiation by withdrawal of CHIR and PMA, single O4 positive cells are interspersed in the neural clusters, as identified by TUBBIII positive neurons. (B) Following reseeding as single cells and recovery of one more week, single O4/OLIG2 double positive oligodendrocytes can be identified. It is likely that this overall efficiency was diminished by the poor survival of oligodendrocytes to replating, which was necessary for the experiment. (TIF) [file pone.0059252.s002.tif]

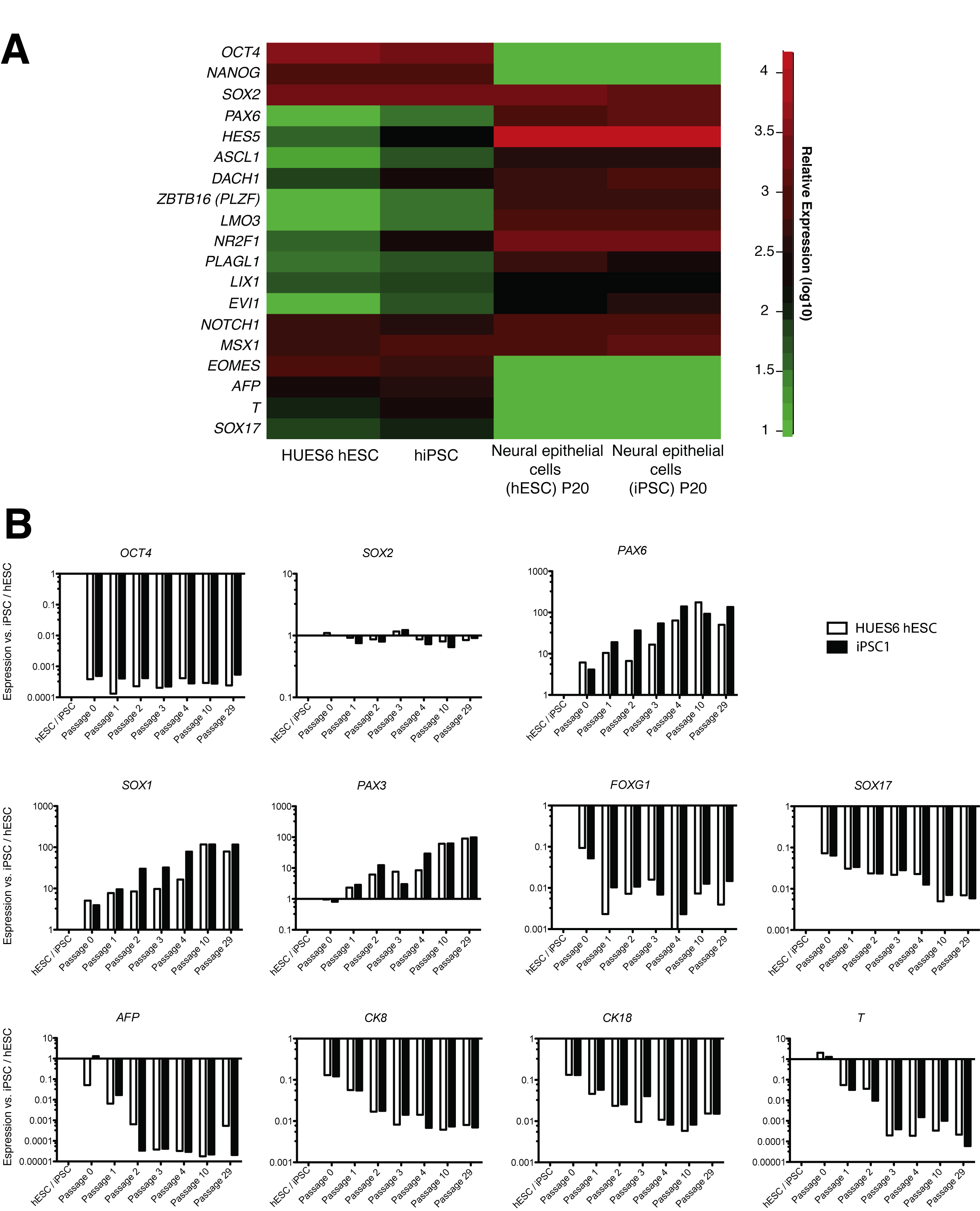

Supplement: Figure S3 — Gene expression by neural epithelial cells. (A) Expression levels for the indicated genes derived from microarray analysis of 2 neural epithelial cell lines and their parental human pluripotent cell lines. Neural epithelial cells consistently expressed neural progenitor and rosette markers, but not markers of pluripotency or mesendodermal differentiation. (B) qRT-PCR analysis of 2 indicated neural epithelial cell lines at the indicated passage number for the indicated gene. OCT4, SOX2 = pluripotency markers. SOX2, SOX1, PAX6 = neural progenitor markers. PAX3 = neural plate border marker. FOXG1 = anterior neural progenitor marker. AFP, SOX17 = endodermal markers. T, CK8, CK18 = mesodermal markers. Microarray data has been deposited in NCBI under accession number GSE40556 and can be accessed through the link: http://www.ncbi.nlm.nih.gov/geo/query/acc.cgi?token=zzijjqwuasuugxe&acc=GSE40556 (TIF) [file pone.0059252.s003.tif]

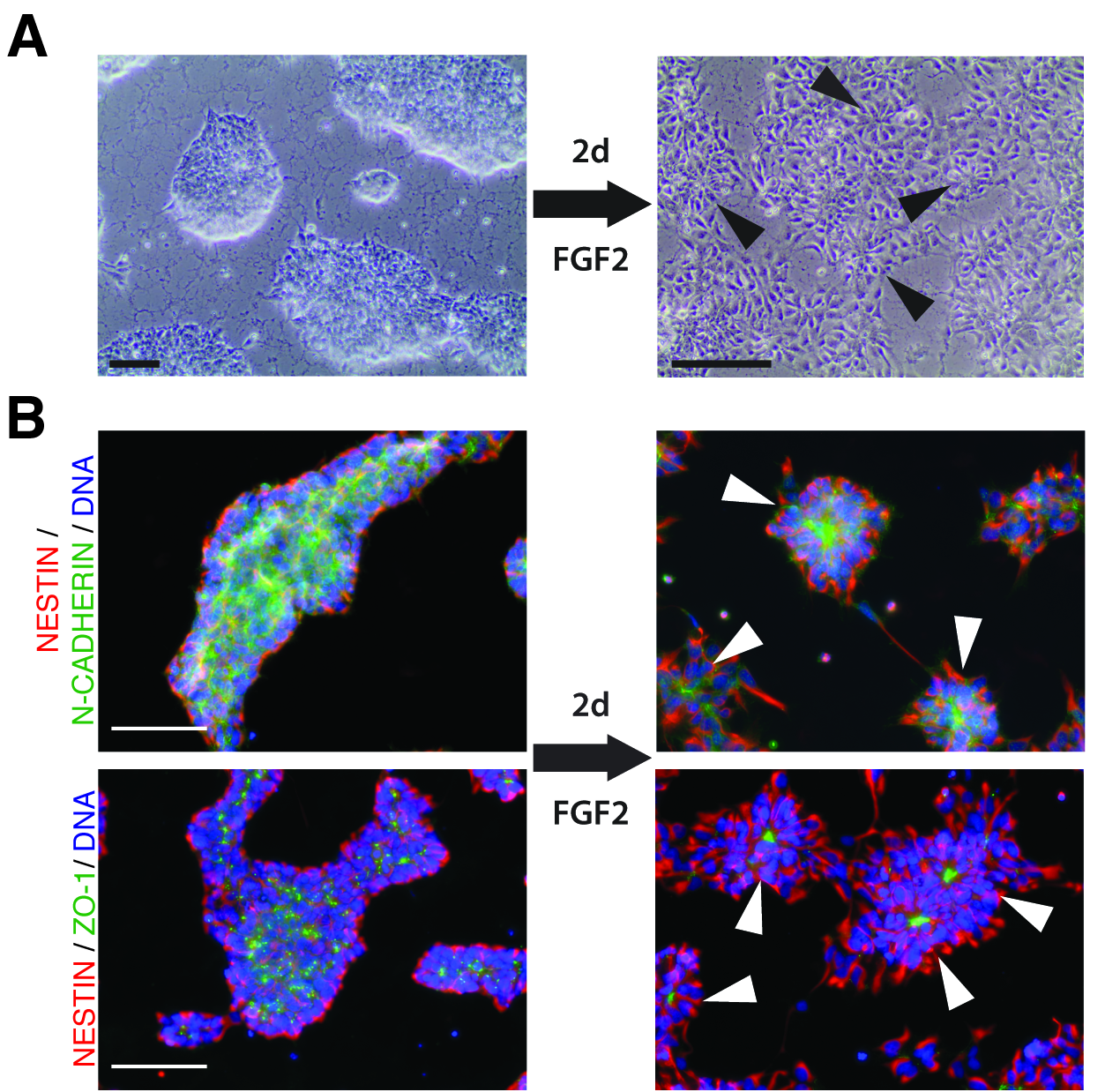

Supplement: Figure S4 — Neural epithelial cells are capable of forming neural rosettes. (A) Phase-contrast image of smNPCs before and after treatment with FGF2 for 2 days. (B) Immunostaining of neural epithelial cells before and after treatment with FGF2 for NESTIN, N-CADHERIN, and ZO-1. Arrowheads indicate N-CADHERIN and ZO-1 expression in the center of neural rosette-like structures, in contrast to the diffuse expression in neural epithelial cells. Scale bars are 100 µm. (TIF) [file pone.0059252.s004.tif]

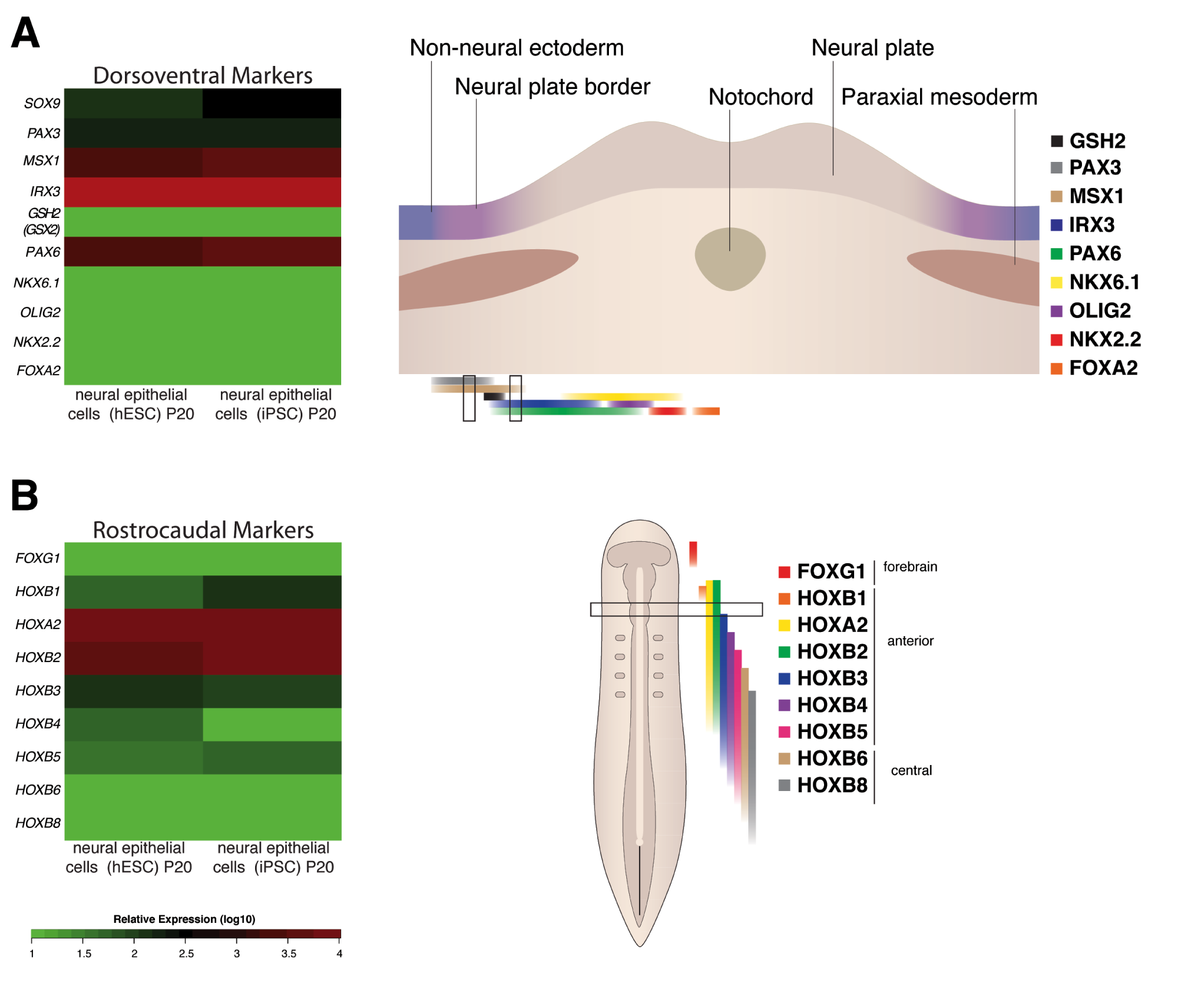

Supplement: Figure S5 — Neural epithelial cells express markers of a moderately dorsal, hindbrain character. Microarray data of the indicated neural epithelial cell lines for markers of dorsoventral (A) and rostrocaudal (B) patterning. A schematic representation of the expression patterns of these markers in vivo in the developing neural tube is shown to the right. The black box indicates the approximate position of neural epithelial cells. See also Figure S2. (TIF) [file pone.0059252.s005.tif]

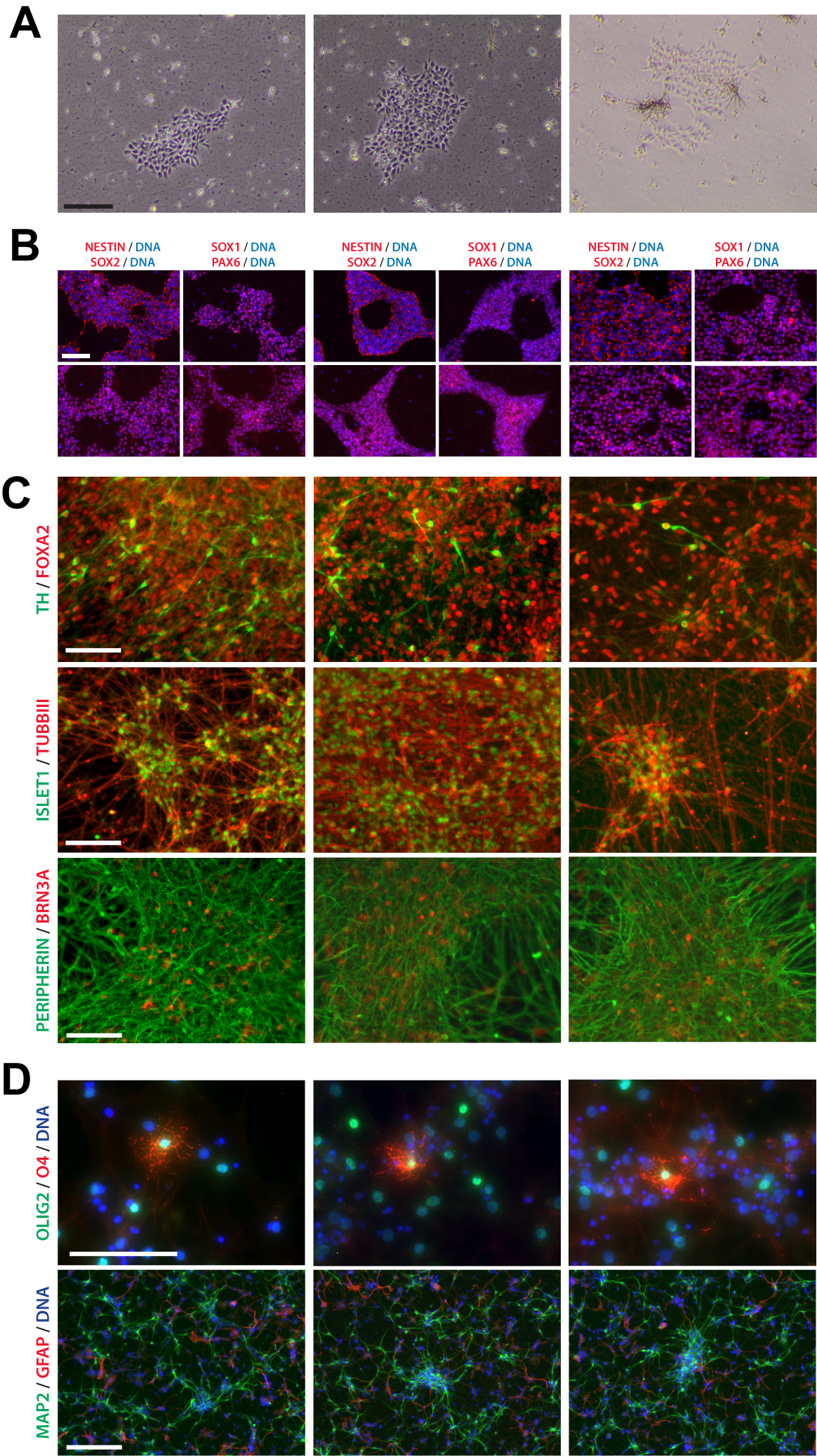

Supplement: Figure S6 — Differentiation of neural epithelial cells into neural crest progenitors. (A) qRT-PCR of neural epithelial cells after treatment with CHIR or BMP4 for 6 days for the neural crest markers PAX7 and SOX9 and TFAP2A. (B) Immunostaining for TFAP2A after differentiation of neural epithelial cells with CHIR for 2 days followed by BMP4 for two days, indicating a strong increase in TFAP2A positive cells. (C) After maturation, PNS-differentiated cells give rise to PERIPHERIN/TFAP2A double – positive cells. (C) Confocal imaging demonstrating BRN3A and PERIPHERIN double-positive peripheral neurons. (D) Immunostaining of primary human fibroblasts (hFib) or neural epithelial cell–derived mesenchymal cells differentiating and cultured with serum for the indicated mesenchymal markers. (E) After 14 days of differentiation with the Human Mesenchymal Stem Cell Functional Identification Kit (R&D Systems), neural epithelial cell–derived cells were assessed for alkaline phosphatase activity and OSTEOCALCIN expression, which are markers of osteocytes, as well as for cells containing oil droplets with FABP4 expression, which is a marker of adipocytes. Scale bars are 100 µm. (TIF) [file pone.0059252.s006.tif]

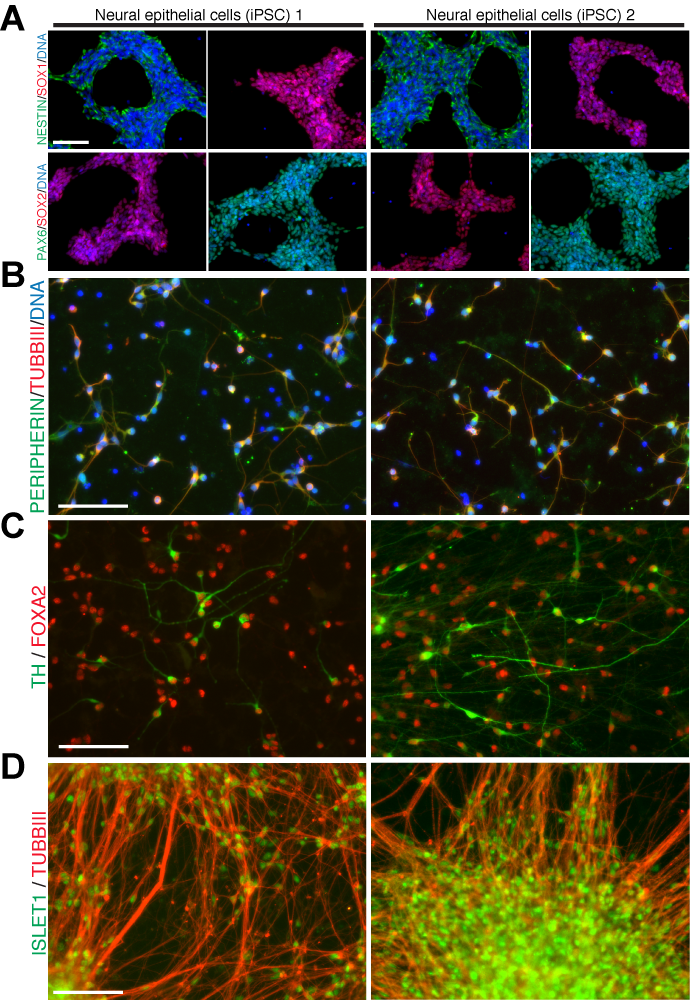

Supplement: Figure S7 — Clonal differentiation into CNS and PNS lineages. (A) Phase-contrast images of 3 independent single cell–derived neural epithelial cell colonies that were picked and expanded. (B) Immunostaining of the resulting clonal neural epithelial cell lines for the indicated neural progenitor markers. (C) Immunostaining for mDANs (TH/FOXA2), MNs (ISLET1/TUBIII), and PNS sensory (PERIPHERIN/BRN3A) neurons from the 3 clonal neural epithelial cell lines. (D) Clonally derived lines are multipotent and can give rise to neurons (MAP2), astrocytes (GFAP) and oligodendrocytes (O4/OLIG2) by spontaneous differentiation. Scale bars are 100 µm. (TIF) [file pone.0059252.s007.tif]

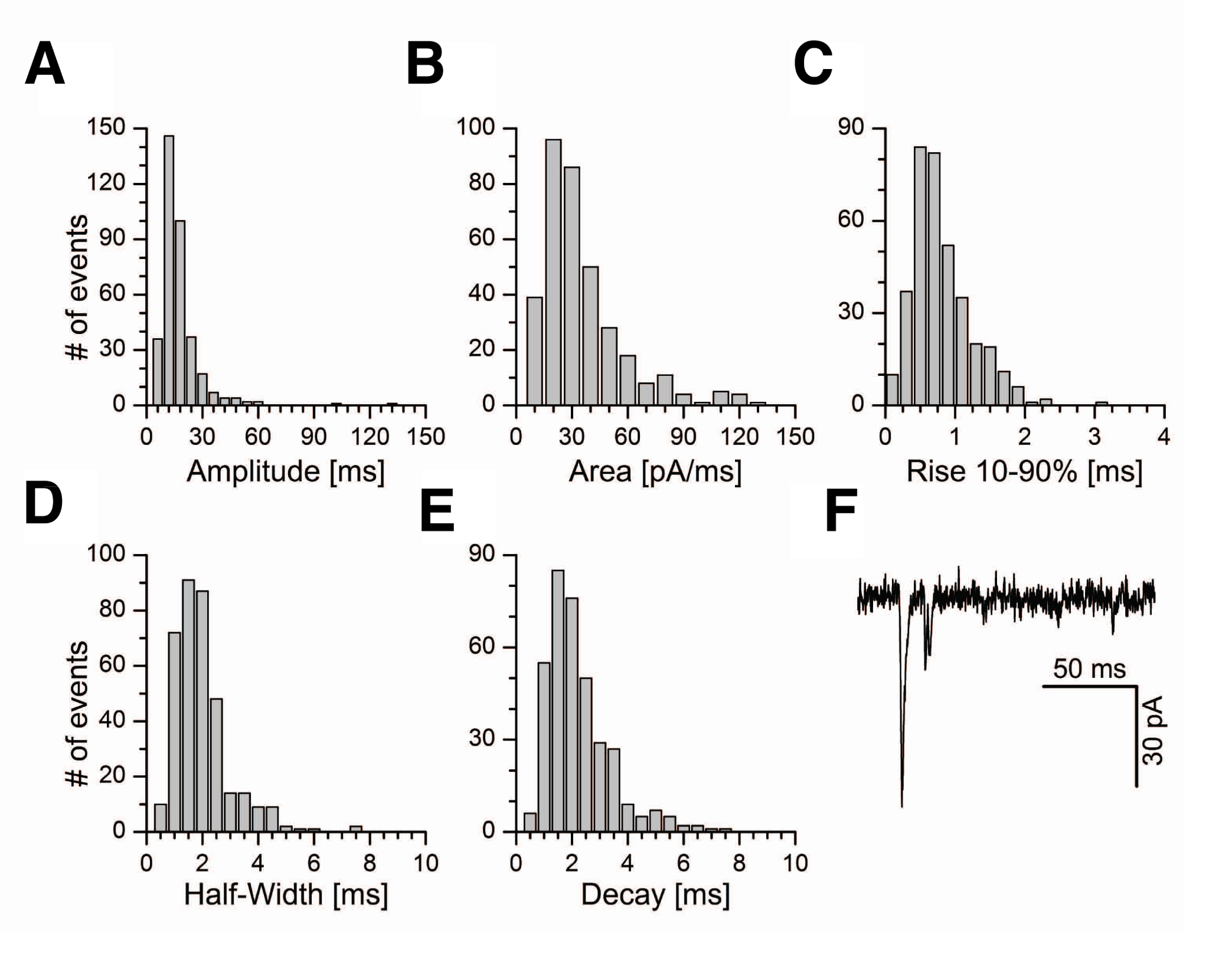

Supplement: Figure S8 — smNPC-derived neurons acquire excitable properties of neuronal cells. (A–E) Amplitude and kinetic parameters (n = 7 cells) and an exemplary recording of minis (F) performed at holding potential −70 mV after two to three weeks of maturation. (TIF) [file pone.0059252.s008.tif]

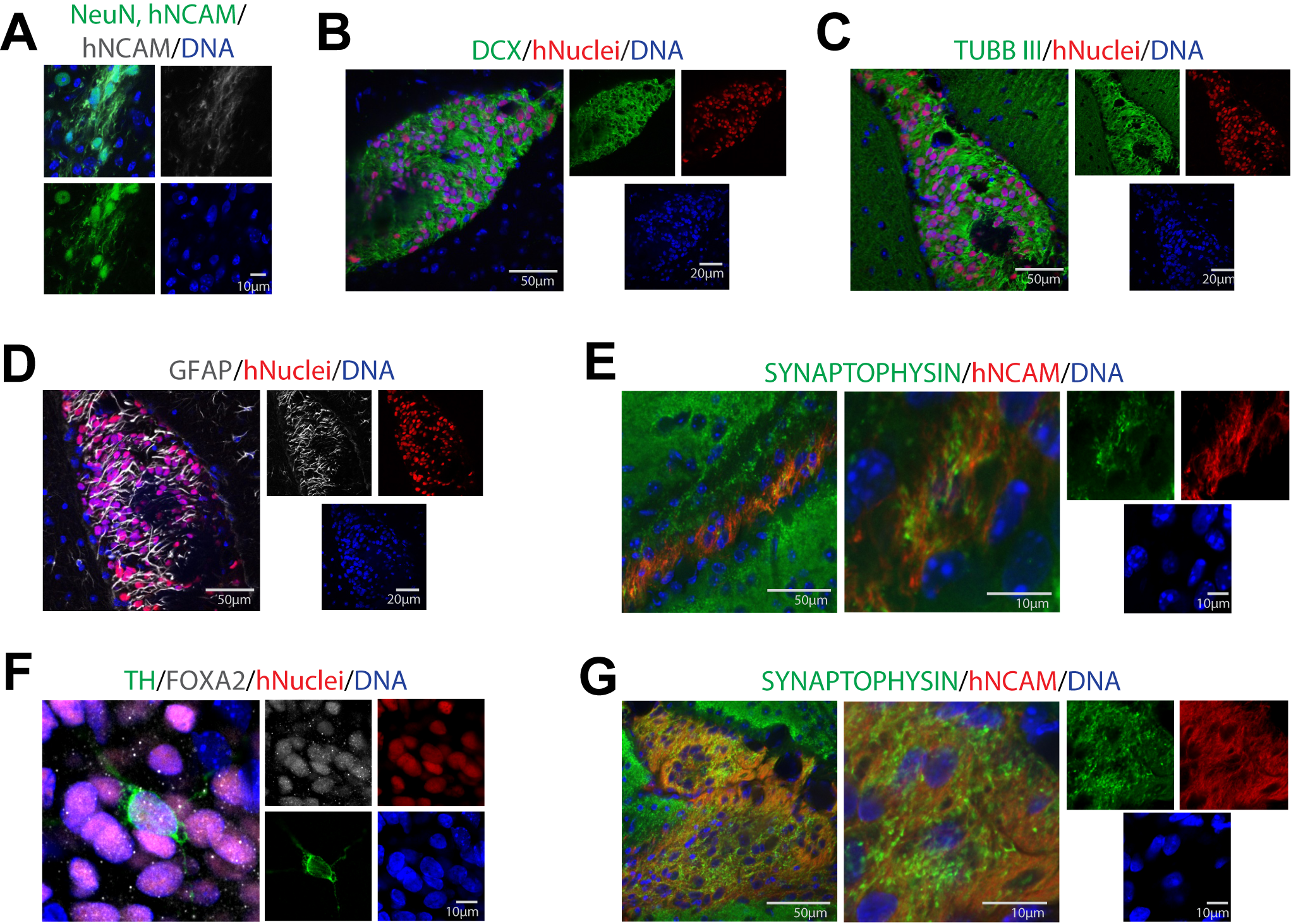

Supplement: Figure S9 — In vivo differentiation potential and long-term survival of transplanted smNPCs. (A) Already after two weaks of maturation in vivo, smNPCs form mature, postmitoctic neurons, as shown by NeuN and human – specific NCAM staining. (B–E) Eight weeks post transplantation, smNPCs have formed and survived as neurons and astrocytes, as shown by staining for DCX, TUBBIII and GFAP and form multiple synapses, as shown by staining for SYNAPTOPHYSIN. (F) After eight weeks, smNPCs prepatterned to an mDAN fate give rise to TH and FOXA2 double positive cells. Without prepatterning, cells are negative for FOXA2 (not shown). (G) Also mDAN-prepatterned smNPCs form synapses as shown by SYNAPTOPHYSIN staining. (TIF) [file pone.0059252.s009.tif]

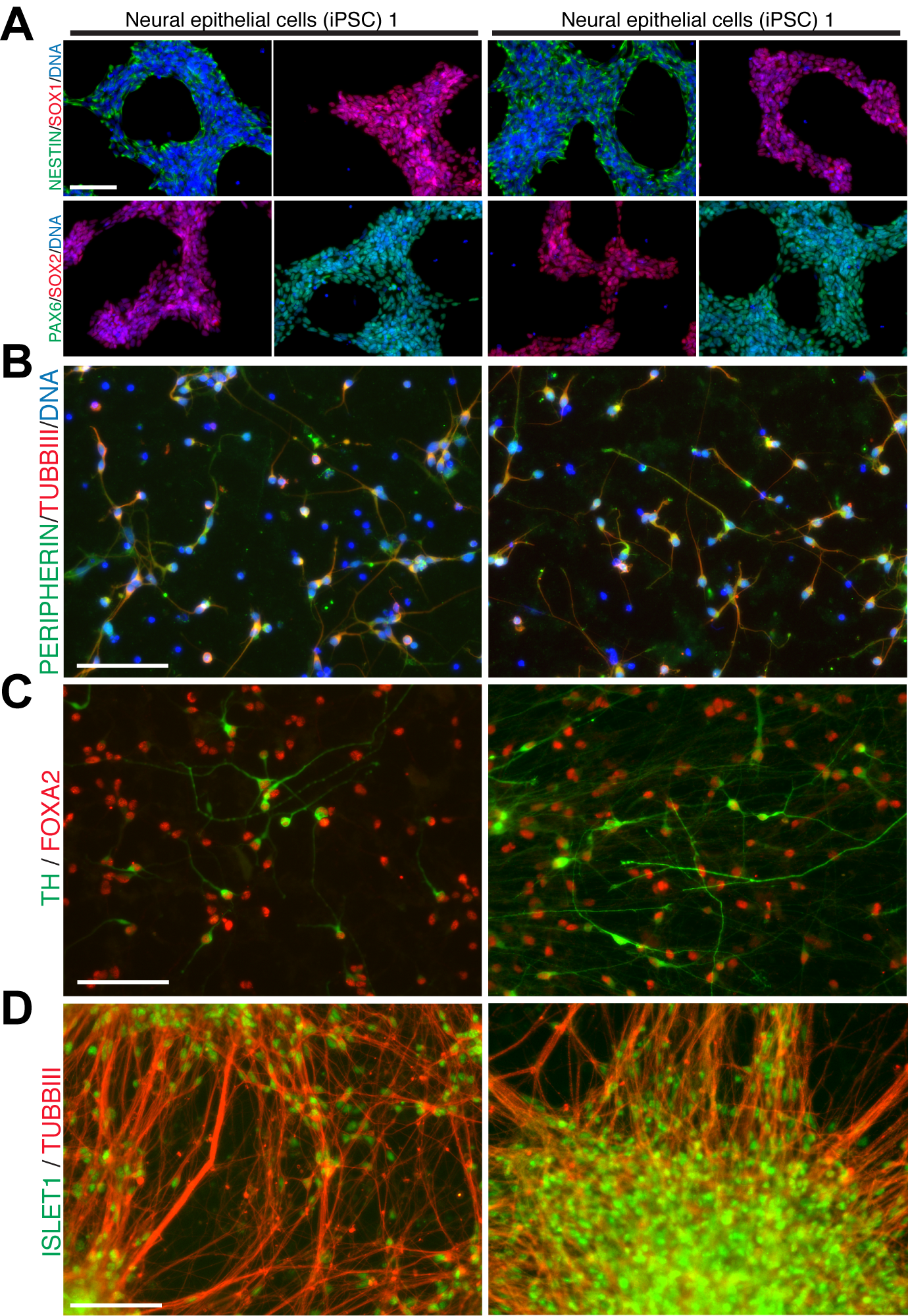

Supplement: Figure S10 — Human iPSC-derived smNPCs. 2 independent smNPC lines were derived from iPSCs. The results of all experiments with hiPSC-derived smNPCs were directly comparable to those with hESC-derived smNPCs. (A) Immunostaining results for the indicated neural progenitor markers. Immunostaining of PNS neurons (B), mDANs (C), and MNs (D) differentiated from the iPSC-derived smNPCs. Scale bars are 100 µm. (TIF) [file pone.0059252.s010.tif]

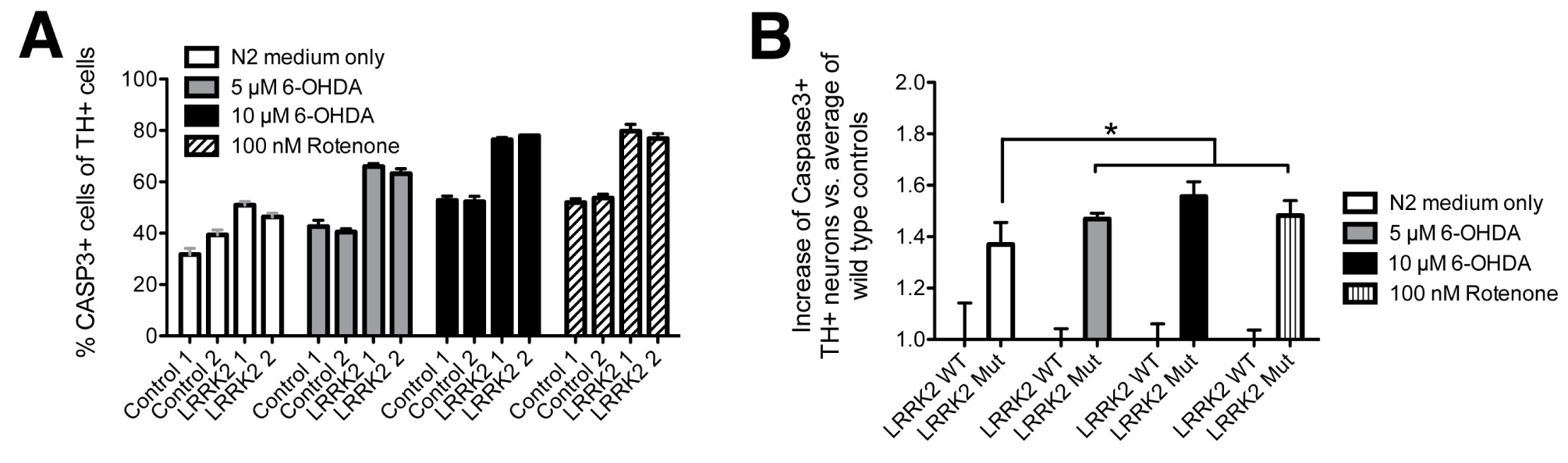

Supplement: Figure S11 — LRRK2 G2019S increases sensitivity of dopaminergic neurons derived from smNPCs to stress compared with controls. smNPCs were derived from two patient-specific LRRK2 mutant iPSCs, alongside with two age- and sex-matched controls. smNPCs were differentiated into mDANs, replated as single cells and incubated with N2 medium only, or supplemented with 5 µM 6-Hydroxydopamine (6-OHDA), or 10 µM 6-OHDA, or 100 nM rotenone. After two days, apoptotic mDANs were identified by immunostaining for TH and cleaved CASPASE3 (CASP3). Error bars represent variation between two independently stressed wells. (A) LRRK2 mutant mDANs show a higher degree of apoptosis in TH+ cells, as compared to healthy controls. (B) Additional stressors separate the mDAN cytotoxicity phenotype better between LRRK2 mutant and wild-type neurons than withdrawal of antioxidants and neurotrophins alone. *indicates p<0.05 according to the Student’s t-test. (JPG) [file pone.0059252.s011.jpg]

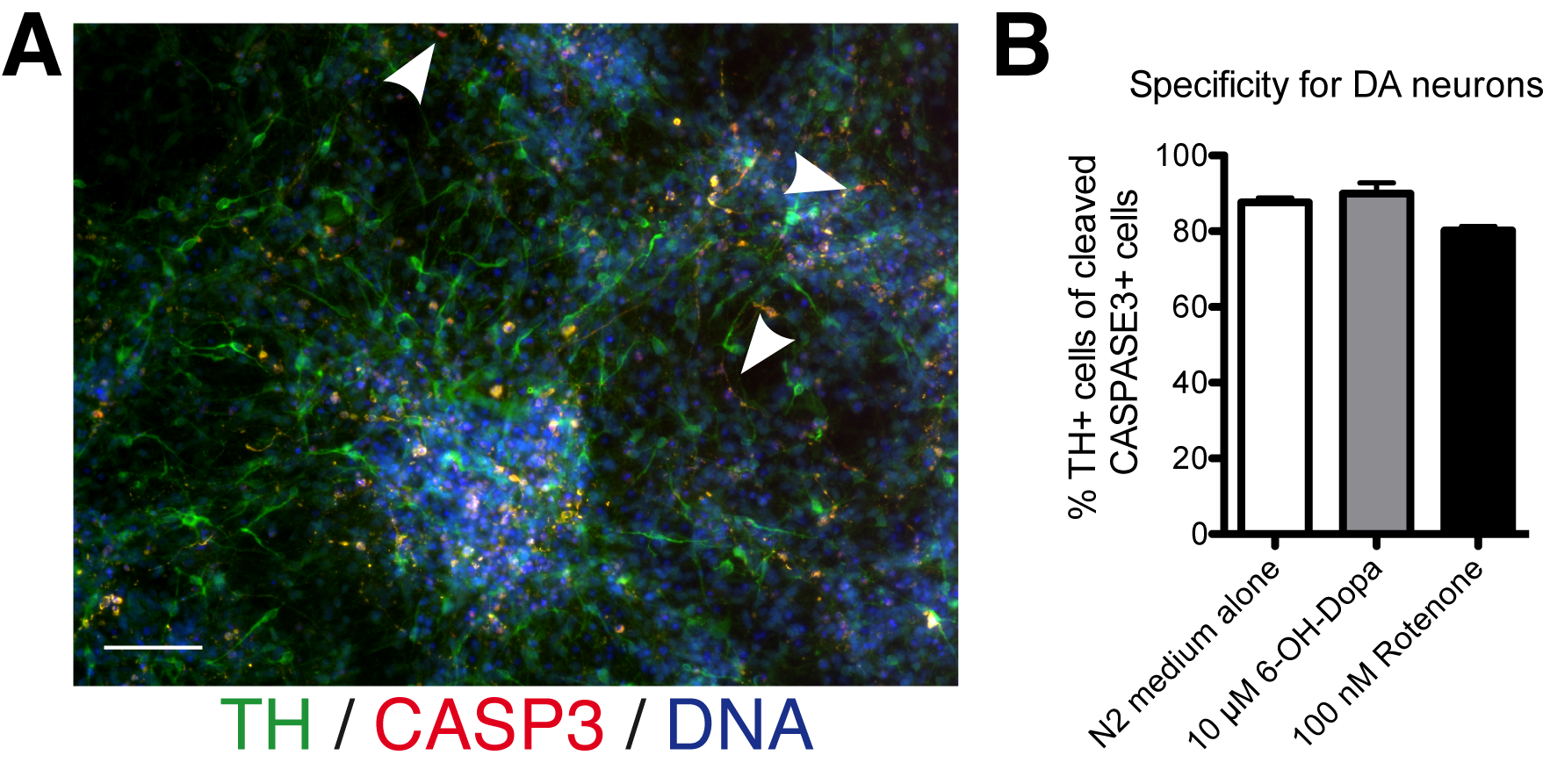

Supplement: Figure S12 — smNPC-derived mDANs are specifically susceptible to oxidative stress. (A) Overview image of a stressed dopaminergic neuron culture after differentiation from smNPCs stressed with 6 OH-Dopa for two days before fixation. Cultures were stained with the indicated markers. Note that most of the cells positive for cleaved CASPASE3 (CASP3) as an indicator for apoptosis are also positive for TH. Only few CASP3+ cells are negative for TH (some indicated by arrowheads). The scale bar indicates 100 µm. (B) Stressed cultures were stained for TH and CASP3 and counted. The graph indicates a high specificity of stress-induced apoptosis for dopaminergic neurons as shown by CASP3 and TH double-positive cells. Under the tested conditions, more than about 80% of the CASP3-positive cells also express TH. The experiment was performed in duplicates. Error bars indicate variance. (TIF) [file pone.0059252.s012.tif]
