# Supplementary material for: Derivation and Expansion Using Only Small Molecules of Human Neural Progenitors for Neurodegenerative Disease Modeling
Source: PLoS One. 2013 Mar 22;8(3):e59252. doi: 10.1371/journal.pone.0059252 (PMC3606479; doi:10.1371/journal.pone.0059252)
Supplement: Table S1 — Primers used for qRT-PCR used in this study. (DOCX) [file pone.0059252.s013.docx]

**Table S1. Primers used for qRT-PCR in this study.**

| **Gene** | **Forward primer (5’-3’)** | **Reverse primer (5’-3’)** |
| --- | --- | --- |
| ***ACTB*** | TCAAGATCATTGCTCCTCCTGAG | ACATCGCTGGAAGGTGGACA |
| ***GAPDH*** | CTGGTAAAGTGGATATTGTTGCCAT | TGGAATCATATTGGAACATGTAAACC |
| ***OCT4*** | GGAAGGAATTGGGAACACAAAGG | AACTTCACCTTCCCTCCAACCA |
| ***SOX2*** | TGGCGAACCATCTCTGTGGT | CCAACGGTGTCAACCTGCAT |
| ***MSX1*** | CGAGAGGACCCCGTGGATGCAGAG | GGCGGCCATCTTCAGCTTCTCCAG |
| ***PAX3*** | AGAGATCACCTCTTGCTTGAGAACG | GGAGCCTGTGCTGTAGCAATCA |
| ***GLI2*** | AAAAGGCCTCTCCTTTGGTGG | ATGGTACCTTCCTTCCTGGTG |
| ***NKX2.1*** | CGTACCAGGACACCATGAGG | GGGCCATGTTCTTGCTCAC |
| ***NKX6.1*** | GCCTCGGAGAACGAGGAAGA | CGCTGCTGGACTTGTGCTTC |
| ***FOXA2*** | CCATGCACTCGGCTTCCAG | TGTTGCTCACGGAGGAGTAG |
| ***OLIG2*** | GCAGAGCACTGCACTTGACTTCTT | CTTCTGCAGTGCGGTTCCTG |
| ***SOX1*** | CAGCAGTGTCGCTCCAATTCA | GCCAAGCACCGAATTCACAG |
| ***HOXA2*** | GCTCGCTGAGTGCCTGACAT | GGAGGAGGAATCAGTGTCGAGTG |
| ***HOXB2*** | GCCGACTCCTGTCTCCAGCTAT | ACTGCAGGTCGATGGCACAG |
| ***HOXA4*** | CAGCAGGTCTTGGAGCTGGA | TCTTGACCTGGCGCTCAGAC |
| ***HOXB4*** | CTACTGCCGCTGCTGGAAGA | TGTGTGTGTGTTACCGTGACCAA |
| ***HOXB8*** | GTCGGCTTCGAGATCTTCTTCG | GATTCGCCGGCTCCTAGTCA |
| ***HOXA9*** | CGTGCAGCTTCCAGTCCAAG | GTGGTGGTGATGGTGGTGGT |
| ***PERIPHE-***  ***RIN*** | TCTGATCAAGACCATTGAGACCC | TCAGTAACTGTGGGCAGAAGAC |
| ***BRN3A*** | GTACCCGTCGCTGCACTC | GGCTTGAAAGGATGGCTCTTG |
| ***EN1*** | AACCGCTACATCACGGAGCA | GATCTTGGCGCGCTTGTTCT |
| ***LMX1A*** | CAACTCAACAGAGGCGAGCATT | GTTTTGGAACCACACCTGGAC |
| ***LMX1B*** | ATCGTGGCCATGGAACAGAG | GTCTGAGGAGCCGAGGAAG |
| ***NURR1*** | TCGACATTTCTGCCTTCTCCTG | GGTTCCTTGAGCCCGTGTCT |
| ***AADC*** | TGCGAGCAGAGAGGGAGTAG | TGAGTTCCATGAAGGCAGGATG |
| ***CHAT*** | GCAGAGCAGCAGGAAGCTGA | TTCTGCCGAGGAGGCTAAGTG |
| ***HB9*** | TGCTTCAGGAGCAGCAAACAA | GATCCAACGCCCTTCCAGAG |
| ***ILSET1*** | AAGCAGCCGGAGAAGACCAC | TCCTTCATGAGCGCATCTGG |
| ***AFP*** | CTGCAAACTGACCACGCTGGAAC | TTCAAGAGGGTTTTCAGTCTGGA |
| ***CK18*** | CAACGGGATCCTGCTGCACCTTG | ATCCGGCGGGTGGTGGTCTTTTG |
| ***PAX6*** | CCAGCCAGACCTCCTCATAC | TGGCTGACTGTTCATGTGTGTC |
| ***FOXG1*** | TGGGAGATAGGAAAGAGGTGAAAA | GCACCAGGCTGTTGATGCT |
| ***SOX17*** | TTTCATGGTGTGGGCTAAGGACG | TGGGGTGGTCCTGCATGTGCTG |
| ***CK8*** | GAAGGGCTGACCGACGAGATCAA | CCAGCCAGGCTCTGCAGCTCC |
| ***T*** | TCTCCCTLCCCTCCACGCACAG | GCCGTTGCTCACAGACCACAGG |
| ***TFAP2a*** | ACCTCGAAGTACAAGGTCACG | GATCTTCCTCCATTTTTAGACTTCG |
