# Supplementary figures and images for: Correction: Derivation and Expansion Using Only Small Molecules of Human Neural Progenitors for Neurodegenerative Disease Modeling
Source: PLoS One. 2013 Nov 14;8(11):10.1371/annotation/6a917a2e-df4a-4ad9-99bb-6aa7218b833e. doi: 10.1371/annotation/6a917a2e-df4a-4ad9-99bb-6aa7218b833e (PMC3838740; doi:10.1371/annotation/6a917a2e-df4a-4ad9-99bb-6aa7218b833e)

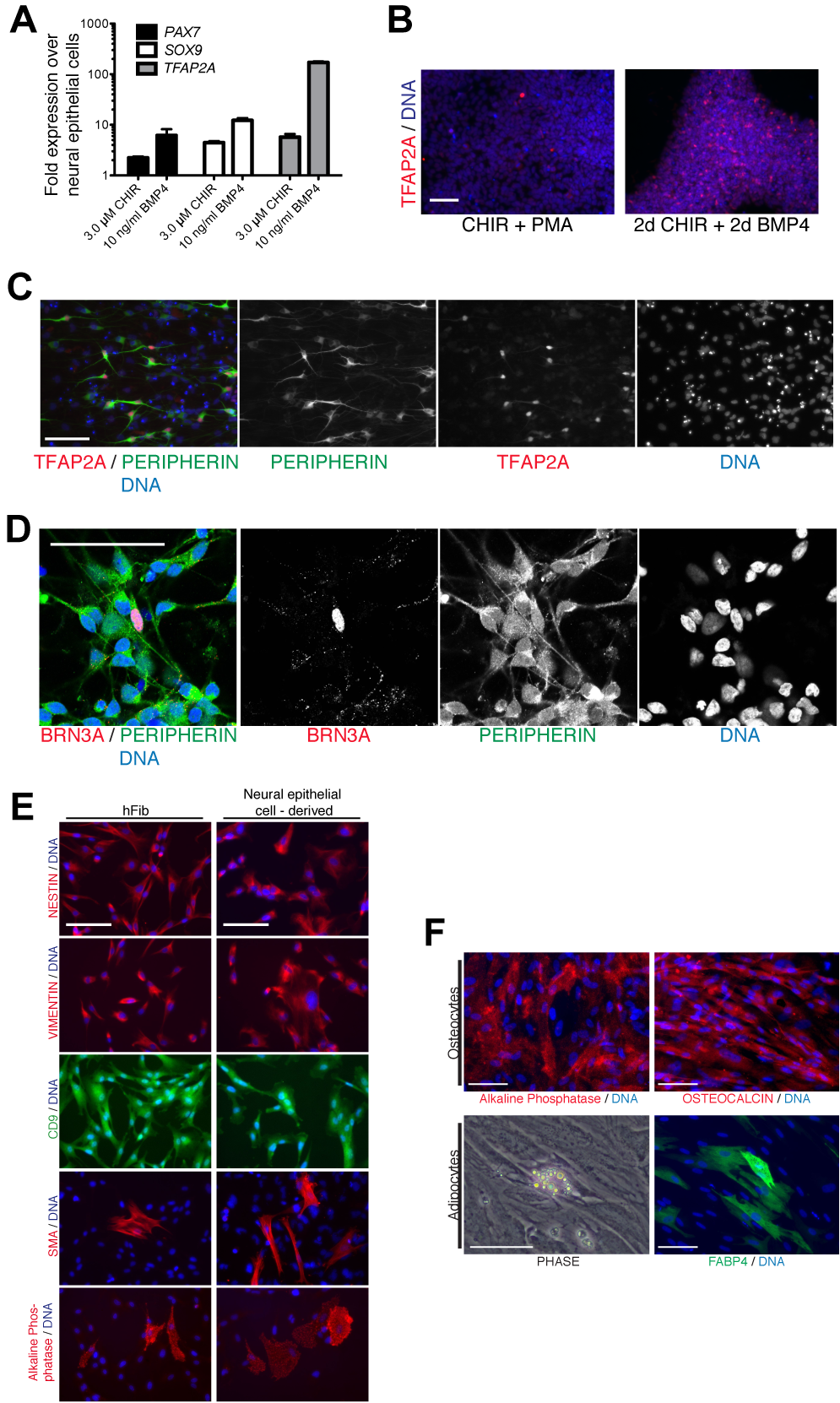

Supplement: Supplementary file 1 [file pone.6a917a2e-df4a-4ad9-99bb-6aa7218b833e.s001.tif]

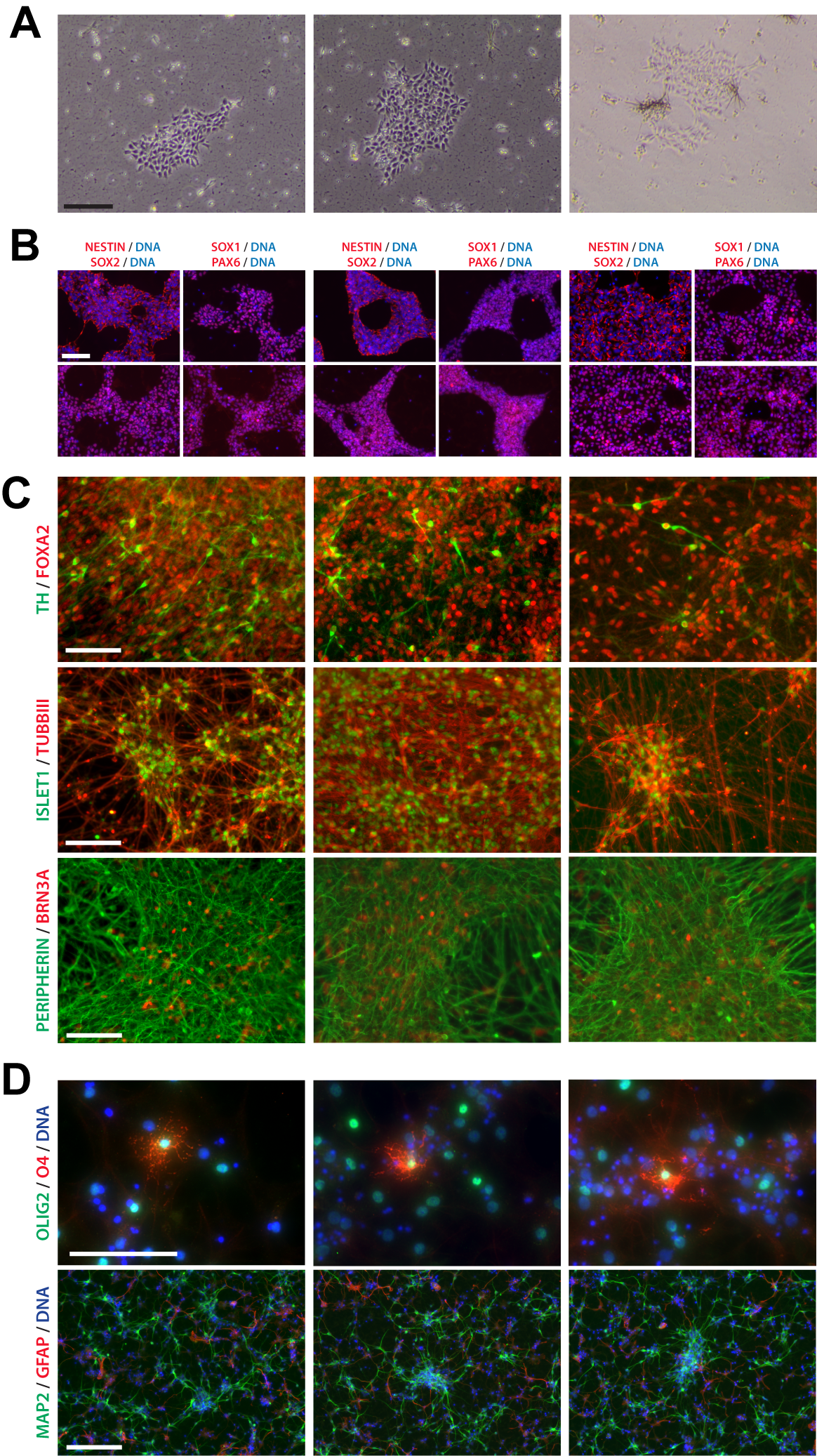

Supplement: Supplementary file 2 [file pone.6a917a2e-df4a-4ad9-99bb-6aa7218b833e.s002.tif]
